# Design and Evaluation of Synthesized Pyrrole Derivatives as Dual COX-1 and COX-2 Inhibitors Using FB-QSAR Approach

**DOI:** 10.1021/acsomega.3c06344

**Published:** 2023-12-12

**Authors:** Shoruq Ahmed Naji, Begüm Nurpelin Sağlik, Mariangela Agamennone, Asaf Evrim Evren, Nalan Gundogdu-Karaburun, Ahmet Çagrı Karaburun

**Affiliations:** † Faculty of Pharmacy, Department of Pharmaceutical Chemistry, Anadolu University, 26470 Eskişehir, Turkey; ‡ Department of Pharmacy, University “G. d’Annunzio” of Chieti-Pescara, Via dei Vestini 31, 66100 Chieti, Italy; § Vocational School of Health Services, Pharmacy Services, Bilecik Seyh Edebali University, 11230 Bilecik, Turkey

## Abstract

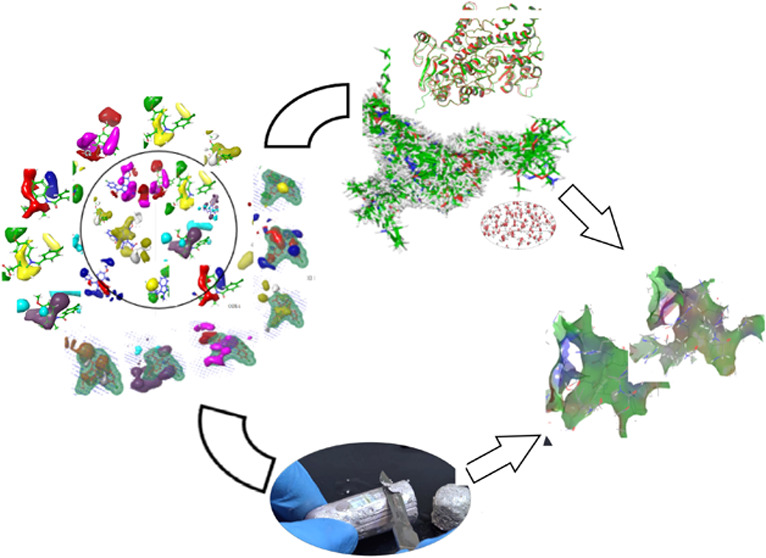

This study delves into the intricate dynamics of the inflammatory response, unraveling the pivotal role played by cyclooxygenase (COX) enzymes, particularly COX-1 and COX-2 subtypes. Motivated by the pursuit of advancing scientific knowledge, our contribution to this field is marked by the design and synthesis of novel pyrrole derivatives. Crafted as potential inhibitors of COX-1 and COX-2 enzymes, our goal was to unearth molecules with heightened efficacy in modulating enzyme activity. A meticulous exploration of a synthesis library, housing around 3000 compounds, expedited the identification of potent candidates. Employing advanced docking studies and field-based Quantitative Structure–Activity Relationship (FB-QSAR) analyses enriched our understanding of the complex interactions between synthesized compounds and COX enzymes. Guided by FB-QSAR insights, our synthesis path led to the identification of compounds **4g, 4h, 4l, and 4k** as potent COX-2 inhibitors, surpassing COX-1 efficacy. Conversely, compounds **5b and 5e** exhibited heightened inhibitory activity against COX-1 relative to COX-2. The utilization of pyrrole derivatives as COX enzyme inhibitors holds promise for groundbreaking advancements in the domain of anti-inflammatory therapeutics, presenting avenues for innovative pharmaceutical exploration.

## 1 Introduction

Inflammation is a vital immune response that defends the body against injuries and infections.^1^ While acute inflammation is crucial for defense, chronic inflammation is linked to diseases like cancer, neurological disorders, and heart disease.^2,3^ COX-1 and COX-2 enzymes convert arachidonic acid into prostaglandins, and NSAIDs inhibit both isoforms, providing anti-inflammatory and analgesic effects.^4,5^ While COX-1 inhibition may lead to gastrointestinal complications, the inhibition of COX-2 is essential for therapeutic effects. To address the challenge of developing safer NSAIDs, researchers are exploring molecular hybridization.^6^ Pyrrole-based NSAIDs, such as tolmetin, ketorolac, and indomethacin, selectively block either COX-1 or COX-2.^7,8^ Pyrrole, known for its distinct reactivity structure, and has become a focal point in medicinal chemistry research, with numerous newly designed anti-inflammatory compounds incorporating at least one heterocyclic ring.^9,10^ Pyrrole serves as a valuable building block in organic synthesis, finding applications in pharmaceuticals, agrochemicals, and natural products.^12,13^

Harrak et al. looked at several compounds related to pyrrole and acetic acid. The one that stopped enzymes the best had an IC_50_ of 5.8 M, which is about 17 times higher than that of ibuprofen.^11^ On the other hand, Kim et al. concentrated on developing compounds with two hydrophobic parts to interact with the lipophilic region within the enzyme’s active site. At the same time, they kept the functional group that interacts with Arg120. This led to lead compound **8n**, which is active against the mPGES-1 enzyme (IC_50_ = 4.5 and 6.9 nM).^12^ Additionally, Park et al. continued this exploration and successfully synthesized a potent phenylsulfonyl hydrazide (**7d**; IC_50_ = 0.06 μM against PGE2). Their focus was on enhancing interactions with Arg120 and other key regions within the enzyme’s active site.^13^ Because of these important results, more research needs to be done on how to make compounds with a good balance of hydrophobic interactions and functional group activity to improve their anti-inflammatory effects.

**Figure 1:**
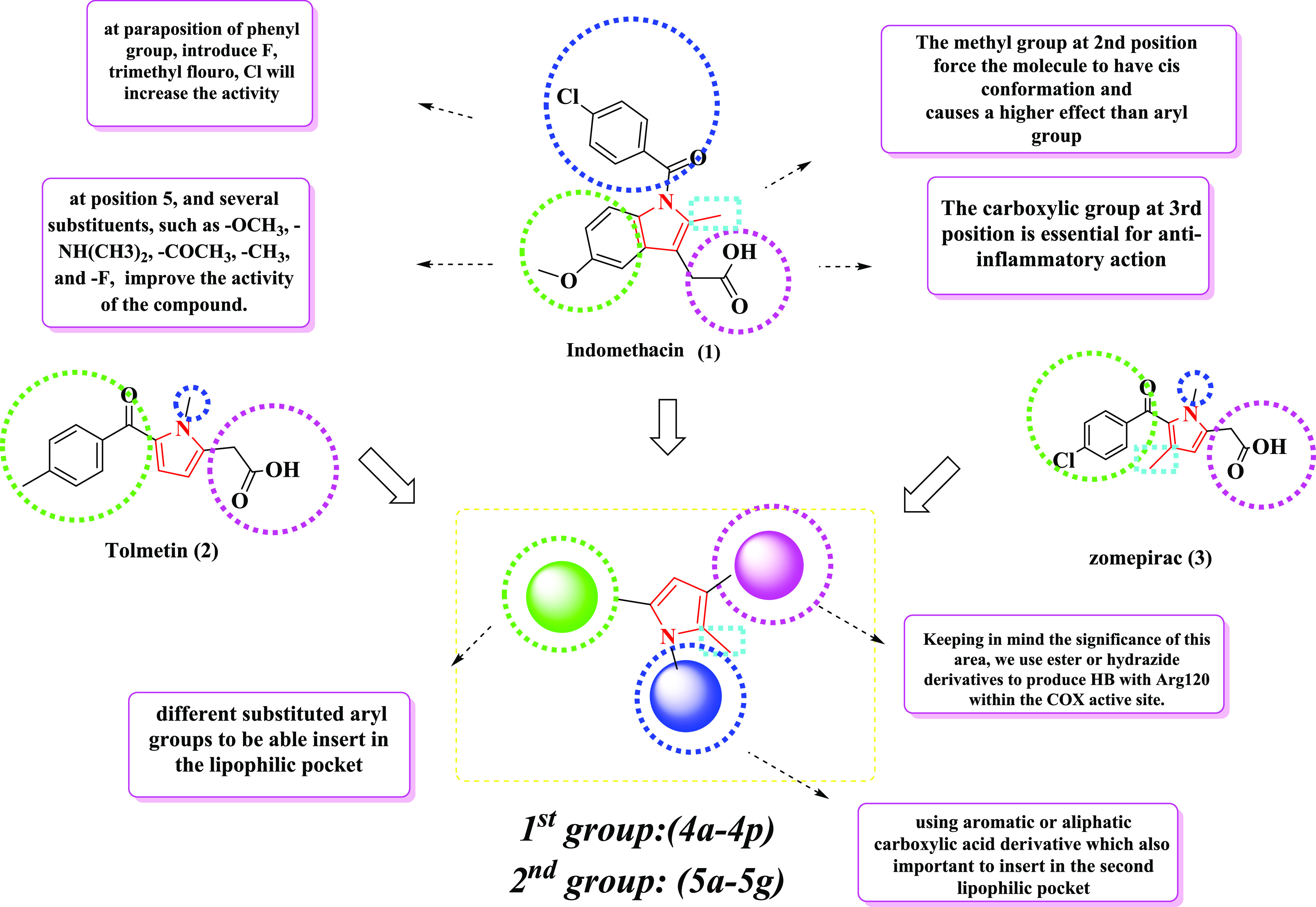
Presentation examples of indomethacin (1), zomepirac (2), tolmetin (3), designed compounds (**4d**–**4p** and **5a**–**5g**), and the development of novel COX inhibitors.

Our research aims to develop cyclooxygenase (COX) inhibitors that strike a balance between the two enzymes, enhancing the efficacy of anti-inflammatory agents.^12,13^ Inspired by the anti-inflammatory potency of zomepirac and tolmetin, and the selective COX-2 inhibition of celecoxib and valdecoxib, we seek to create a new class of anti-inflammatory agents with improved safety profiles.^12,13^ In pursuit of our goal, we employed the field-based quantitative structure–activity relationship (FB-QSAR) approach to design novel *N*-pyrrole carboxylic acid derivatives, strategically incorporating an acidic group to enhance anti-inflammatory effects and leveraging 2-methylpyrrole pharmacophores as potent COX-1 and COX-2 inhibitors. Through docking studies, we gained valuable insights into the anti-inflammatory activity and binding affinity of the synthesized compounds for the COX-1 and COX-2 active sites. This research strategy, accompanied by a tailored data set, focuses on the synthesis of substituted 2-[3-(ethoxycarbonyl)-2-methyl-5-(substituted phenyl)-*1H*-pyrrole-1-yl] alkanoates, as depicted in Figure 1, to evaluate their potential as effective COX inhibitors.^14^ Our multidisciplinary approach, encompassing molecular design, computational modeling, structural analysis, and in vitro assays, propels significant advancements in anti-inflammatory drug discovery with the overarching objective of providing safer and more efficient solutions to inflammation-related health challenges. By gaining valuable insights into the efficacy and interactions of the designed compounds, we aspire to drive progress in the field of anti-inflammatory therapeutics, ultimately benefiting research in developing more potential anti-inflammatory compounds.

## 2 Results and Discussion

### 2.1 Designing Promising New Compounds Using the FB-QSAR Model

**Figure 2:**
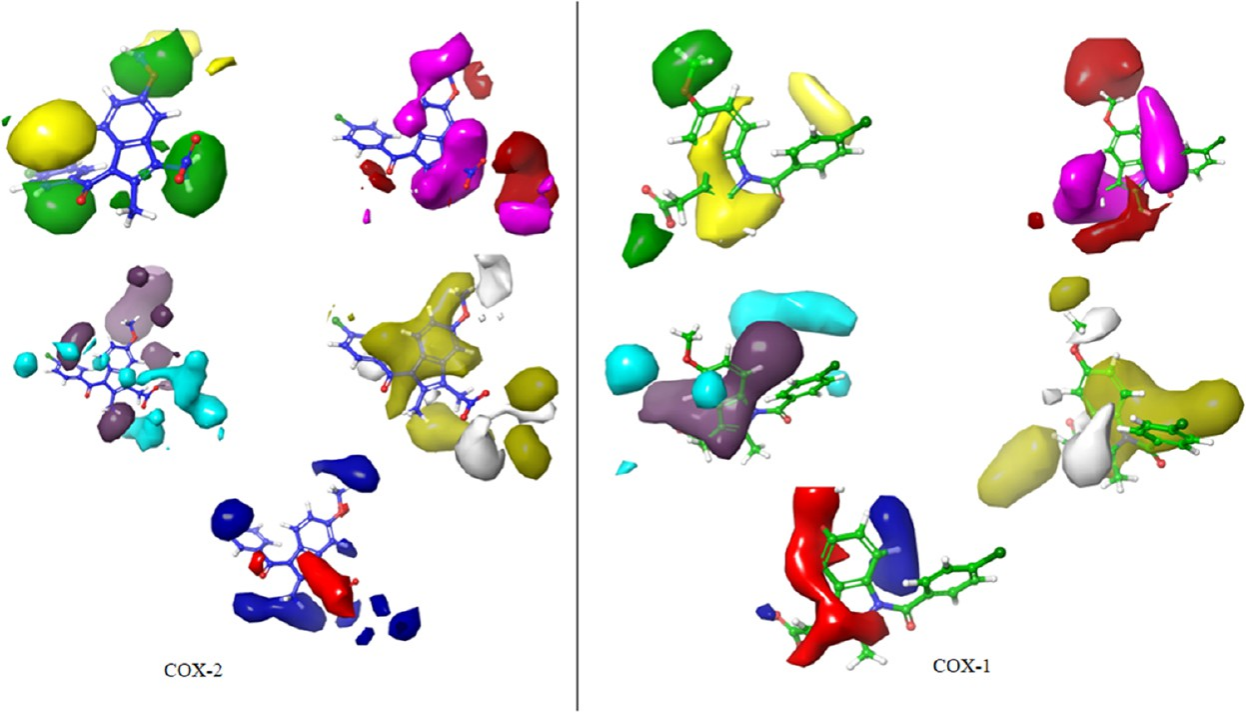
3D Visualizations of indomethacin contour maps COX-2 & COX-1, respectively: steric (positive effect (+): green, negative effect (_): yellow), electrostatic (+: blue, -: red), hydrophobic (+: yellow, -: white), HBA (+:red, -:magenta), and HBD (+:blue-violet, -: cyan), respectively.

Using the data set generated in Section 5.1, we employed the FB-QSAR model to predict the activity of COX-1 and COX-2 inhibitors. The model demonstrated exceptional accuracy and reliability (Figure 2). Analysis revealed that the steric field had the greatest influence on activity, followed by electrostatic and hydrophobic fields. With these insights, we designed 22 promising new compounds guided by the predictions of the FB-QSAR model of high activity. These compounds were selected for synthesis and subsequent testing.

**Figure 3:**
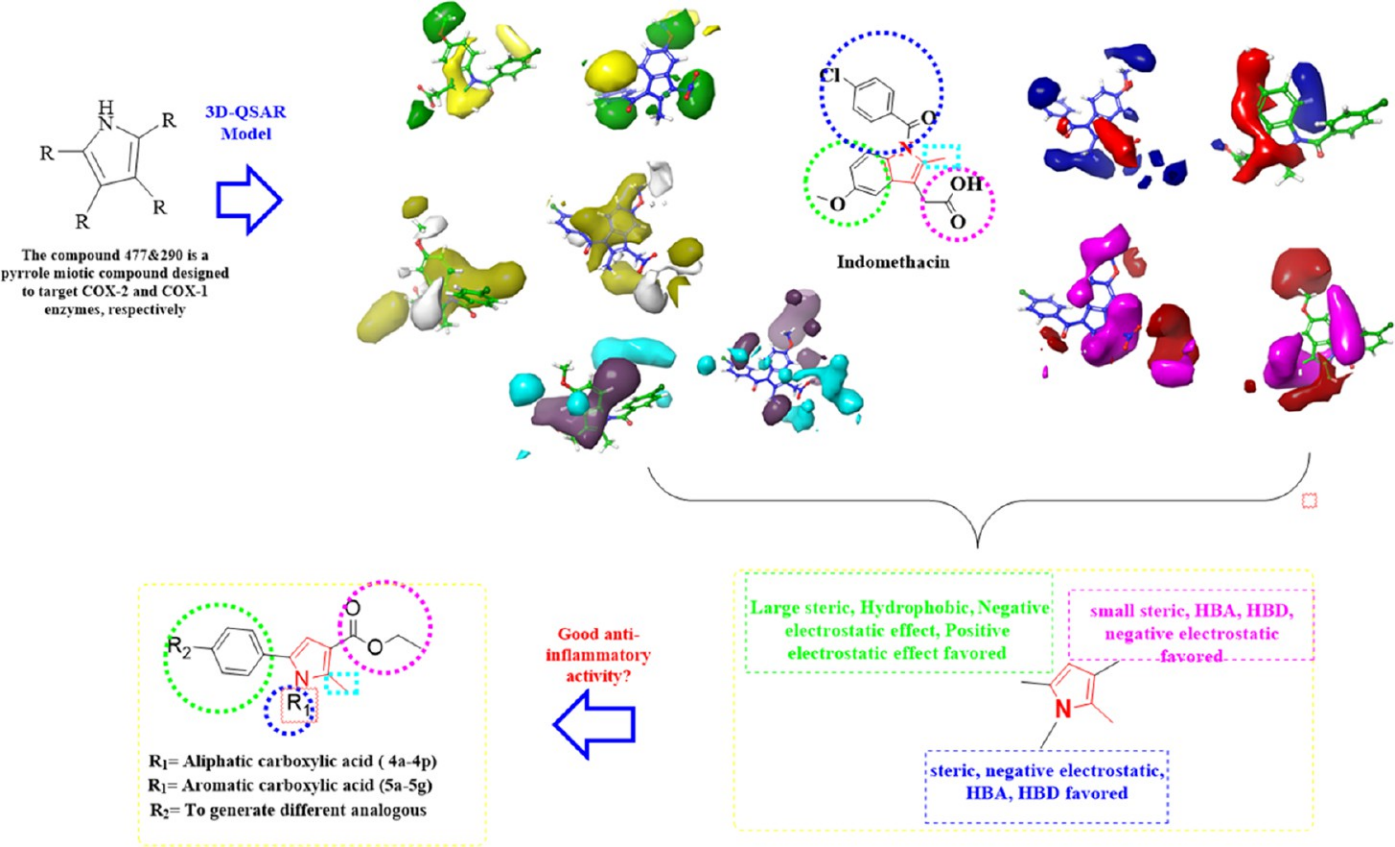
3D-QSAR model-driven design of target compounds (**4a**–**4p** and **5a**–**5g**).

The FB-QSAR model was also utilized to predict the activity of the compounds identified from in vivo studies. Contour maps were generated to investigate the effects of various structural features on the anti-inflammatory activity of indomethacin using a partial least squares (PLS) factor of 5. The analysis of the contour maps provided critical information regarding the impact of different structural modifications on the activity of the synthesized compounds **(4a–4p** and **5a**–**5g**), highlighting positions 1, 3, and 5. At the fifth position of the indole ring, steric groups with negative electrostatic effects were influential. The methyl group at position 2 had a positive electrostatic effect. At the third position, a small steric group and a strongly negative electrostatic group were recommended. Hydrophobic groups at the fourth, fifth, sixth, and seventh positions were advantageous. COX-1 vs COX-2 contour maps showed methoxy’s positive effect on COX-1. Position 2 favored an HBA group and an HBD in the indole ring. Molecular modifications in synthesized compounds (**4a–4p, 5a–5g**) included unchanged methyl groups at position 2, and modifications at positions 1, 3, and 5. Position 1 saw hydrophobic groups with HBA and HBD features added. Position 3 was modified with an ester group acting as an HBA and HBD, maintaining hydrophobic and electrostatic effects. These positions were strategically modified to enhance hydrophobic and electrostatic effects, in line with the FB-QSAR analysis findings (Figure 3).

#### 2.1.1 Evaluation of the FB-QSAR Model in Predicting COX-2 and COX-1 Inhibitor Activity

The reliability of the final FB-QSAR model (referred to as Model 5) in predicting the activity of compounds as COX-2 and COX-1 inhibitors was evaluated using various statistical parameters, including *R*^2^, *Q*^2^, Pearson^–r^, stability, RMSE, *F*, and *P* values. The obtained values for each parameter fell within the acceptable ranges specified in Tables 1 and 2 in the Section 5, indicating the model’s reliability and ability to produce accurate predictions in both internal and external environments. These findings suggest that the model could serve as a valuable tool for medicinal chemists aiming to develop safer and more effective drugs.

**Table 1:** FB-QSAR Model’s Verification Values

	COX-2 INHIBITORS					COX-1 INHIBITORS				
#FACTORS	1	2	3	4	5	1	2	3	4	5
SD	0.5032	0.4138	0.3547	0.3179	0.2905	0.9183	0.6978	0.5523	0.4705	0.4022
*R*^2^	0.6581	0.7700	0.8320	0.8658	0.8886	0.3821	0.6455	0.7793	0.8409	0.8845
stability	0.997	0.992	0.985	0.979	0.969	0.992	0.966	0.936	0.917	0.874
*F*	338.7	293.0	287.2	279.1	274.5	96.5	141.1	181.3	202.2	232.8
*P*	7.17^–43^	1.39^–56^	3.87^–67^	2.66^–74^	5.36^–80^	5.02^–18^	1.26^–35^	2.57^–50^	5.54^–60^	2.46^–69^
RMSE	0.49	0.41	0.37	0.37	0.37	0.73	0.47	0.43	0.41	0.33
*Q*^2^	0.6812	0.7792	0.8192	0.8196	0.8219	0.3087	0.7093	0.7527	0.7800	0.8566
Pearson^–R^	0.8266	0.8845	0.9068	0.9083	0.9090	0.5907	0.8475	0.8784	0.8973	0.9372

**Table 2:** Gaussian (Steric, Electrostatic, Hydrophobic, HBA, and HBD) Effects Observed in the FB-QSAR Results

	COX-2 INHIBITORS	COX-1INHIBITORS
#FACTORS	5	5
steric	0.1948	0.2985
electrostatic	0.1135	0.0948
hydrophobic	0.2270	0.2350
HBA	0.1435	0.1778
HBD	0.1459	0.1938

**Figure 4:**
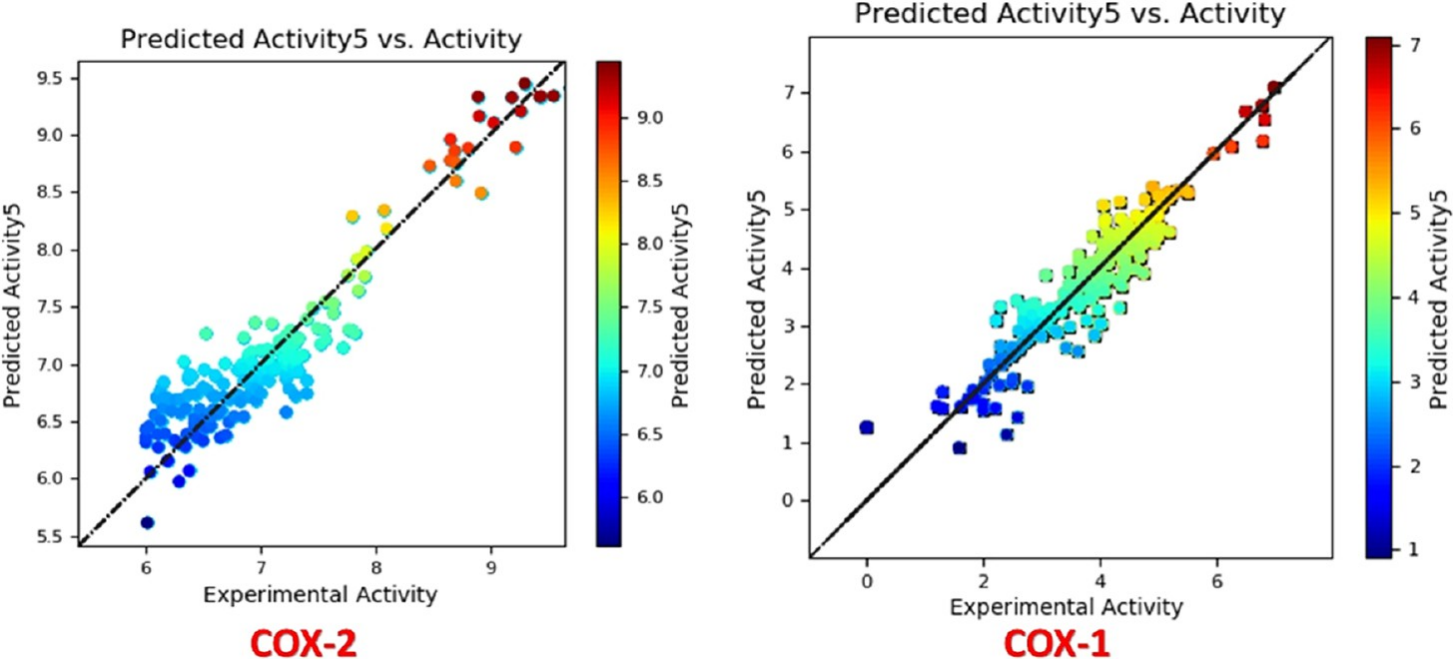
Linear diagrams of the COX-2 FB-experimental QSAR’s and predicted pIC_50_.

**Figure 5:**
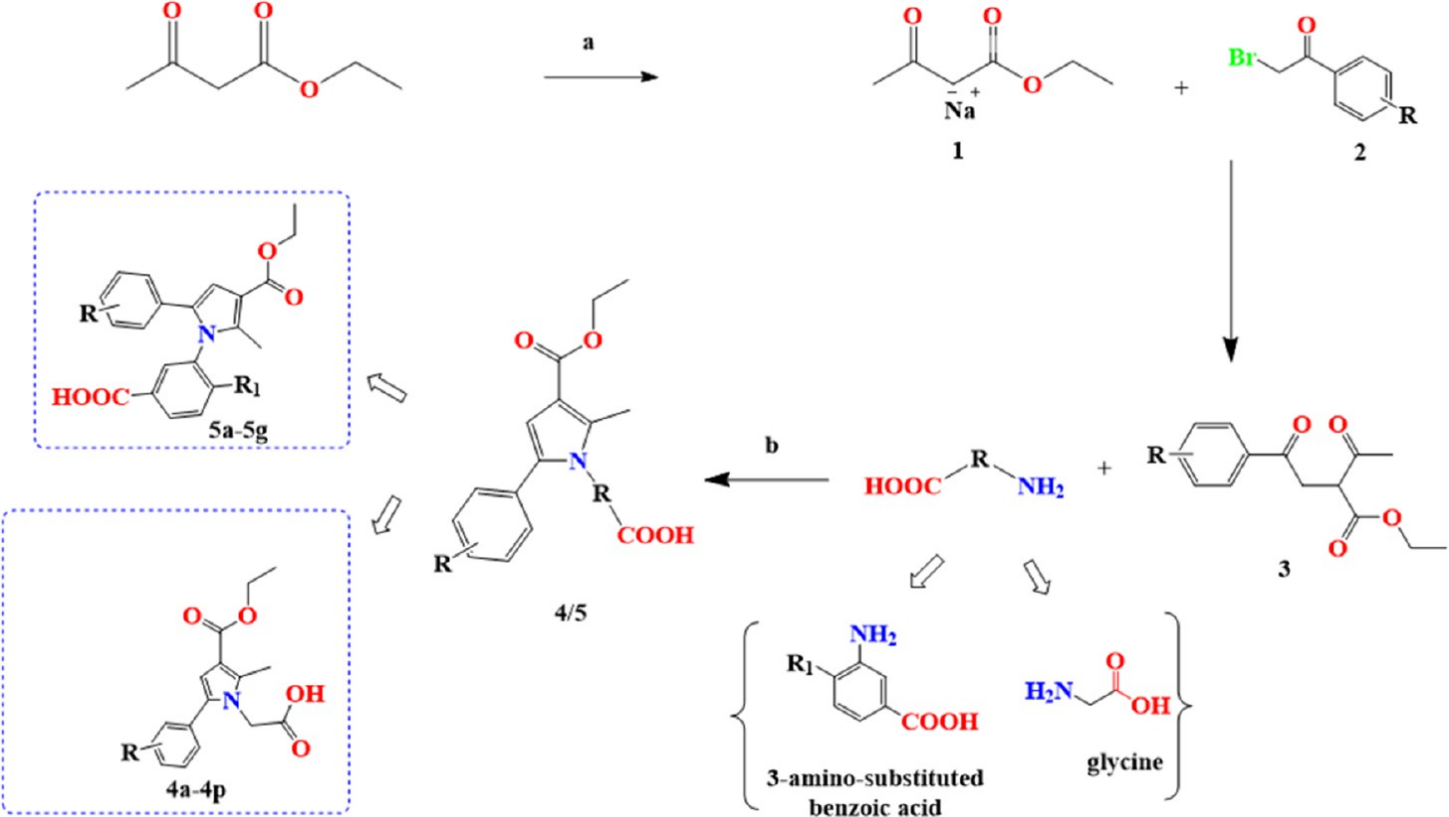
Schematic representation of the synthetic pathways. Reaction conditions: (a) Metallic sodium, anhydrous toluene, r.t; (b) glacial acetic acid, reflux.

The outcomes of the FB-QSAR study are presented in Tables 1 and 2. The findings indicate that the steric field exerted a substantial influence on the inhibitor activity of compounds toward COX-2 and COX-1, contributing to 19.4 and 29.8% of the observed variance, respectively. In contrast, the electrostatic field accounted for 11.3 and 9.4% of the variance in the COX-2 and COX-1 activity, respectively. The CoMSIA model, an extension of the CoMFA model, incorporates additional fields, such as hydrophobic and hydrogen-bond fields. The hydrophobic field was found to exert a substantial effect on the compounds’ COX-2 inhibitor activity, explaining 33.7% of the observed variance, whereas the steric field, electrostatic field, hydrogen bond acceptor field, and hydrogen-bond donor field accounted for 19.9, 15.1, 13.4, and 17.8%, respectively. On the other hand, for COX-1 inhibitor activity, the contributions of the different fields were as follows: the steric field accounted for 29.8% of the observed variance, while the electrostatic field contributed 9.4%. The hydrogen-bond acceptor field, hydrogen-bond donor field, and hydrophobic field accounted for 23.5, 17.7, and 19.3% of the variance, respectively. These findings offer insights into the factors influencing the activity of the COX-2 and COX-1 inhibitors and may aid in the rational design of more potent and selective COX inhibitors. Illustrated in Figure 4 is a linear graph that presents both experimental and predicted COX-2 inhibitor activity values for all compounds. Experimental values were extracted from laboratory tests measuring the inhibition of the COX-2 enzyme and sourced from the Chembl database. Meanwhile, the FB-QSAR model utilized compound chemical structures to generate the predicted values. This graph serves to evaluate the model’s predictive performance, with the *x*-axis depicting experimental values and the *y*-axis showing predicted values. Each data point reflects a single compound, and its position indicates the level of agreement between predictions and experiments, with points closer to the diagonal line representing higher accuracy (Figure 5).

### 2.2 Evaluation of the Synthesis of the Target Compounds

After obtaining the skeleton of our target compound from the FB-QSAR model described in Section 2.1, we opted to focus on 1,2,3,5-tetra-substituted pyrrole derivatives as our selected compounds. The synthetic procedure employed for these compounds involved a meticulously designed four-step pathway utilizing the Paal–Knorr synthesis technique. This method enabled the successful synthesis of pyrrole by utilizing a 1,4-dicarbonyl compound as a precursor for the formation of the pyrrole ring, along with a substituted amine, followed by dehydrative condensation. The process commenced with ethyl 3-oxobutanoate as the initial starting material, and anhydrous sodium metal was introduced as the reducing agent. This catalyzed a series of transformations leading to the formation of radical intermediates, referred to as compound **1**. These intermediates exhibited significant nucleophilic properties, acting as carbanions in search of suitable chemical partners. Subsequently, bromoacetophenone derivatives were added, with the carbonyl groups serving as electrophiles. This resulted in the generation of compound **3**, namely, ethyl 2-acetyl-4-oxo-4-(substituted phenyl)butanoate. Compound **3** showcased increased complexity and sophistication compared to its precursor, reflecting the progress made in the synthesis process. The synthesis journey continued with the introduction of an amino-substituted compound, which underwent cyclization in the presence of acidic conditions, specifically glacial acetic acid. This crucial step led to the formation of a pyrrole ring, adding an elegant dimension to the synthesis and bringing the target compound closer to realization. The synthesis was finalized by quenching the reaction with ice water, which caused the desired target compound **4** to precipitate out of the solution. Undesirable compounds such as NaBr remained in the water phase. This final step ensured the isolation of the target compounds, marking the successful completion of the synthesis process. To confirm the authenticity of the synthesized compounds, rigorous spectroscopic methods, including ^1^H NMR, ^13^C NMR, and HRMS, were employed. These spectral analyses were instrumental in verifying the structures of the obtained derivatives of 2-[3-(ethoxycarbonyl)-2-methyl-5-(substituted phenyl)-*1H*-pyrrol-1-yl]-substituted carboxylic acid (Figures S1–S79).

### 2.3 Evaluation of Chemical Spectra

#### 2.3.1 NMR Analysis Reveals Structural Insights of Synthesized Compounds

^1^H NMR and ^13^C NMR analyses were conducted on the synthesized compounds using a Bruker UltraShield 400 MHz instrument with DMSO-*d*_6_ as the solvent. Substituents on the phenyl ring at position 5 led to distinct chemical shifts in the ^1^H NMR spectra. Methyl groups in the second position had a singlet peak from 2.35 to 2.58 ppm, and C4–H protons appeared as singlet peaks between 6.25 and 6.67 ppm. The adjacent methyl group to the ethoxycarbonyl group appeared as a quartet at 4.15 and 4.26 ppm, while the terminal methyl group was observed as a triplet at 1.24–1.38 ppm. In the first group (**4a**–**4p**) with *N*-pyrrole attached to aliphatic carboxylic acid (acetic acid), the methyl proton signal in acetic acid ranged from 4.05 to 4.73 ppm, aligning with the adjacent methyl of the ester group. In the second group (**5a**–**5g**) with *N*-pyrrole attached to aromatic carboxylic acid (4-substituted-3-amino benzoic acids), specific substituents (−OCH_3_ and −OH) yielded singlet peaks at 3.78 ppm and broad singlet peaks at 11.21 ppm, respectively. The COOH group exhibited a broad singlet peak from 11.19 to 13.09 ppm. Carbonyl carbon in the ester group ranged from 164.59 to 164.96 ppm, while in acetic acid groups, it shifted to 169.89–170.52 ppm. The methyl group in the second position exhibited upfield shifts at 11.5 and 11.79 ppm, whereas the terminal methyl carbons of the ester group were positioned upfield at 14.85–14.99 ppm. And methyl groups adjacent to acetic acid and ester groups displayed downfield shifts (46.34–49.86 and 58.88–59.50 ppm) due to the carbonyl group’s electron-withdrawing nature. All aromatic carbons at position 5 appeared within 112.44 and 158.85 ppm, with identified substitutions causing distinctive shifts. Comprehensive ^13^C NMR and ^1^H NMR values can be found in Figures S87and S88.

### 2.4 Evaluation of Activity

An in vitro study was conducted to evaluate the COX-1 and COX-2 inhibitory properties of pyrrole carboxylic acid derivatives. Results presented in Table 3 demonstrate that all compounds and reference drugs exhibited over 50% inhibition activity at concentrations of 1 and 10 μM, using fluorometric inhibitor screening kits [Section 5.3]. Compounds containing the acetic acid group at position 1 (**4g, 4h, 4k, and 4l**) showed the highest activity against both COX-2 and COX-1, with IC_50_ values indicating greater activity compared to celecoxib. Specifically, compounds **4k** and **4h** demonstrated IC_50_ values against COX-2 and COX-1, respectively. Compounds **4h** and **4g** exhibited higher inhibition against COX-1, comparable to ibuprofen. Conversely, compounds containing a hydroxybenzoic acid substituted at position 1 (**5e** and **5b**) exhibited the most activity against COX-1. The presence of a small acidic group at position 1 proved to be effective against both COX-1 and COX-2 for all synthesized compounds. However, if a larger group was introduced, the activity would become biased toward COX-1. Furthermore, the compounds containing acetic acid moieties at position 1, along with a lipophilic and bulkier group at position 5, showed simultaneous activity against both COX-1 and COX-2. Conversely, when a lipophilic but smaller group was substituted, the activity favored the inhibition of COX-2 inhibition. However, increasing bulkiness at position 1 and replacing the acetic group with a more substantial acidic group shifted the activity back toward favoring COX-1. Overall, the study highlights the significant impact of small chemical structural changes in pyrrole carboxylic acid derivatives on their COX-1 and COX-2 inhibitory activities. These findings provide valuable insights into the design and development of more effective COX-1 and COX-2 inhibitors with improved selectivity and reduced side effects. Future research can focus on optimizing the structure of pyrrole carboxylic acid derivatives to enhance their therapeutic potential.

**Table 3:** Values for IC_50_ (μM) and Percent Inhibition at 10 and 1 μM for Both Synthesized Compounds and Reference Drugs against COX-1 and COX-2

	COX-1% Inhibition		COX-1 IC_50_ (μM)	COX-2% Inhibition		COX-2 IC_50_ (μM)
compounds	10 μM	1 μM		10 μM	1 μM	
**4c**	46.122 ± 0.822	32.720 ± 0.957	>10	79.429 ± 2.056	38.749 ± 0.848	>1
**4d**	63.568 ± 1.390	39.447 ± 1.055	>1	47.036 ± 0.961	29.642 ± 0.722	>10
**4e**	74.919 ± 1.862	43.367 ± 1.677	>1	80.441 ± 1.837	48.320 ± 0.836	>1
**4g**	92.366 ± 2.731	84.517 ± 2.036	0.117 ± 0.005	90.967 ± 2.022	83.033 ± 1.878	0.188 ± 0.008
**4n**	41.552 ± 0.921	28.627 ± 0.745	>10	38.748 ± 1.057	21.590 ± 0.967	>10
**4k**	86.461 ± 1.716	46.378 ± 1.501	>1	94.674 ± 2.123	88.055 ± 1.927	0.108 ± 0.004
**4h**	96.628 ± 2.020	92.710 ± 2.674	0.068 ± 0.003	95.374 ± 2.046	90.521 ± 2.459	0.091 ± 0.004
**4i**	82.275 ± 2.355	48.367 ± 2.157	>1	87.759 ± 1.936	38.521 ± 2.241	>1
**4l**	41.326 ± 1.057	34.587 ± 0.836	>10	91.579 ± 2.458	82.602 ± 2.061	0.143 ± 0.006
**4m**	46.259 ± 0.923	26.874 ± 0.755	>10	38.626 ± 0.861	30.855 ± 0.858	>10
**5f**	71.737 ± 1.839	43.330 ± 0.874	>1	79.761 ± 1.963	48.237 ± 1.064	>1
**5e**	93.185 ± 2.031	86.799 ± 1.879	0.129 ± 0.005	74.618 ± 1.884	37.217 ± 0.936	>1
**5g**	85.418 ± 2.416	41.218 ± 1.036	>1	89.176 ± 1.822	37.418 ± 0.874	>1
**5a**	88.038 ± 2.856	47.611 ± 1.802	>1	41.366 ± 1.631	38.044 ± 1.161	>10
**5b**	95.766 ± 2.012	91.499 ± 1.547	0.082 ± 0.003	83.403 ± 2.784	40.498 ± 1.634	>1
**5d**	40.369 ± 0.861	30.748 ± 1.058	>10	48.317 ± 1.361	25.679 ± 0.963	>10
**5c**	48.513 ± 1.236	48.513 ± 1.236	>10	79.501 ± 1.922	79.501 ± 1.922	>1
**SC560**	99.076 ± 2.165	96.274 ± 1.864	0.006 ± 0.0002			
Ibuprofen	98.152 ± 1.058	89.361 ± 1.245	2.450 ± 0.135	98.234 ± 1.208	88.155 ± 1.348	5.326 ± 0.218
celecoxib				92.327 ± 1.425	85.485 ± 1.303	0.132 ± 0.005
nimesulide				97.821 ± 1.214	89.575 ± 1.049	1.684 ± 0.079

#### 2.4.1 FB-QSAR Models Predict the Activity of Structurally Synthesized Compounds (**4a**–**4p** and/**5a**–**5g**)

The primary objective of this study was to employ FB-QSAR models in order to predict the activity of structurally modified compounds (**4a**–**4p** and **5a**–**5g**) and reference anti-inflammatory drugs, with a specific emphasis on their COX-2 and COX-1 inhibitory activity. The compiled data in Table 3 facilitated a comparison between the experimental and predicted pIC_50_ values, utilizing the ideal model, factor 5, for predictions. The results of the study revealed that several synthesized compounds exhibited significantly higher inhibitory activity against COX-2 compared to the reference drugs. For instance, compound **4h** exhibited a predicted pIC_50_ value of 7.11, while compound **4m** demonstrated a pIC_50_ value of 6.62. Both of these values surpassed those of ibuprofen and nimesulide, which were recorded at 6.44 and 6.20, respectively. Notably, all of the benzoic acid derivatives exhibited lower predicted and experimental activity toward COX-2 in contrast to the reference drugs. These findings indicate that the acetic acid analogs displayed greater activity toward COX-2, which was further supported by the results obtained through fluorometric methods employed to measure the activity.

Additionally, among the compounds with benzoic acid substitutions in the first position (**5a**–**5g**), compounds **5b** and **5e** emerged as the most active, showcasing noteworthy COX-1 inhibitory activity. Specifically, compound **5b** exhibited a predicted activity value of 6.80, closely aligned with the experimental value of 7.08. Similarly, compound **5e** demonstrated an experimental activity value of 6.4, which agreed well with the predicted value of 6.8. These results underscore the potent inhibitory potential of these compounds against COX-1. Moreover, Table 4 presents experimentally validated pIC_50_ values obtained using BioVision’s kit protocol for COX-2 inhibitory activity Osmaniye, 2023 #394. Among the reference drugs, celecoxib displayed the highest predicted pIC_50_ value of 7.26, followed by ibuprofen (6.44) and nimesulide (6.20). The experimentally measured pIC_50_ values for celecoxib, nimesulide, and ibuprofen were 6.87, 5.77, and 5.27, respectively, validating the reliability of our model, as there was good agreement between the experimental and predicted values.

**Table 4:** Active Compounds against Inflammation Predicted by FB-QSAR and Confirmed Experimentally

compounds	predicted activity** on COX-2	experimental activity* on COX-2	predicted activity** on COX-1	experimental activity* on COX-1
**4h**	7.1136	7.0411	7.0381	7.16
**4k**	5.9243	6.9665	6.6425	>6
**4l**	6.1436	6.8446	7.0537	>5
**4g**	6.0261	6.7258	5.5803	6.93
**5b**	5.5926	>6	6.8023	7.08
**5e**	5.5925	>6	6.4288	6.88
**5f**	5.9612	>6	3.5327	>6
**4c**	5.4850	>6	6.5773	>5
**4i**	6.1678	>6	6.9314	>6
**5g**	5.3411	>6	7.0381	>6
**4e**	6.1800	>6	6.8358	>6
**4m**	6.6225	>5	5.7777	>5
**4n**	6.0661	>5	5.7076	>5
**5d**	5.9243	>5	5.2097	>5
**5a**	5.5925	>5	6.7074	>6
**4d**	6.4691	>5	6.6342	>6
**5c**	5.3848	>6	5.8383	>5
celecoxib	7.2610	6.87		
nimesulide	6.2041	5.77		
ibuprofen	6.4498	5.27	5.9851	5.61

* Rapid and Reliable Test for Identifying COXs (1 and 2) Enzymes Inhibiting Compounds (BioVision’s kit protocol).

** Prediction activity using FB-QSAR model PLS factor 5.

To summarize, this study demonstrated the potential of FB-QSAR models for predicting the activity of novel compounds for drug discovery. The synthesized compounds exhibited promising COX-2 inhibitory activity, with certain compounds even surpassing the activity of the reference drugs. The experimentally validated pIC_50_ values further supported the accuracy and reliability of the FB-QSAR model in predicting COX-2 inhibitory activity. These findings highlight the significance of utilizing FB-QSAR models in drug development and underscore their potential in the field of COX-2 inhibition.

### 2.5 Evaluation of the Pharmacokinetic Profile

The assessment of the pharmacokinetic profile, including absorption, distribution, metabolism, excretion, and toxicity (ADMET) properties, is a crucial step in identifying promising molecules for drug development. In this study, QikProp software was utilized to predict the pharmacokinetic properties of selected structures, encompassing absorption, distribution, metabolism, and elimination. Ten criteria, linked to the inflammatory process and the reference compound indomethacin, were employed for this evaluation (refer to Table 5). The #star parameter compared the obtained results with the properties of drugs already present in the QikProp software’s database.^31^ When a result fell outside the 95% confidence interval of values comparable to those of commercially available drugs, an alert was triggered. This parameter considered various characteristics, including molecular weight (*M*_W_), dipole moment, number of rotatable bonds (#rotor), number of hydrogen-bond donor groups (HBD), number of hydrogen-bond acceptor groups (HBA), predicted water/gas partition coefficient (QPlog *P*w), predicted octanol/water partition coefficient (QPlog *P*o/w), predicted aqueous solubility (log *S*), prediction of binding to humans, and predicted brain/blood partition coefficient (QPlog BB).^32^ The outcomes for the selected compounds are listed in Table 5. One vital parameter used in drug design to predict solubility, membrane permeability, and compound bioavailability is QPlog *P*o/w, which represents the apparent permeability between octanol and water.^33^ Calculated Qlog *P*o/w values for compounds **4n**, **4i**, **4h**, and **5b** were higher than the value observed for indomethacin (QPlog  *P*o/w = 4.267). For the remaining compounds, the values ranged from 2.695 to 4.306, indicating a higher lipophilicity (log *P*o/w ≥ 0). Consequently, it can be inferred that the new compounds primarily undergo passive transcellular mechanisms for absorption in the intestine.^34^ Furthermore, the central nervous system (CNS) activity of the selected compounds was predicted by using a parameter ranging from −2 (inactive) to +2 (active). Similar to the reference compound indomethacin, compounds **4p**, **4b**, **4c**, **4m**, **4i**, **4n**, **5g**, **5c**, and **5a** exhibited values of (−1), indicating a lower level of activity compared to the maximum level. This suggests that the CNS activity of these compounds is somewhat less than optimal or desired, but not completely inactive.^32^ Another significant parameter, percent human oral absorption (PHOA), predicts the potential oral absorption of a compound on a scale of 0 to 100%. It is closely correlated with Human Oral Absorption, as they measure the same property. A value above 80% indicates high predicted oral absorption, while a value below 25% suggests poor predicted oral absorption. Remarkably, all of the compounds analyzed exhibited values higher than 80%, indicating a high predicted oral absorption. Notably, compounds **4n** (96.06%), **5d** (93.35%), **4i** (93.04%), **4g** (91.71%), and **4k** (91.13%) demonstrated the highest absorbability. Moreover, these compounds exhibited higher values than the reference compound indomethacin (91.87%), which is known for its oral absorption efficiency. To assess the compounds’ compliance with Lipinski’s Rule of Five (RO5), key characteristics such as molecular weight (*M*_W_), lipophilicity (expressed by the partition coefficient, log *P*), and hydrophilicity (determined by the number of hydrogen bond donor and acceptor groups) were considered.^30^ The predicted values indicate that the compounds’ *M*_W_ and log *P* fall within the ranges of (287–452) and (2.695–5.834), respectively. The number of hydrogen-bond donor groups (HBD) and hydrogen-bond acceptor groups (HBA) are both ≤2, with HBA values ≤5.75. Furthermore, all compounds contained in our data set had less than seven rotatable bonds, as required by Lipinski’s Rule of Five. These results suggest that the synthetic compounds can be therapeutically manipulated and utilized in oral drug delivery systems. Except for compounds **5c** and **5g**, no compounds in this study violated Lipinski’s Rule of Five (RO5), indicating that these two compounds are likely to exhibit oral activity in humans. This suggests a similarity to orally administered compounds in terms of biological activity.^35^ Overall, the evaluation of the pharmacokinetic profile using in silico methods proved valuable in prioritizing potential drug candidates. The predicted ADMET properties, Qlog *P*o/w values, CNS activity, PHOA, and adherence to Lipinski’s Rule of Five collectively provide insights into the potential therapeutic viability and oral absorption efficiency of the studied compounds.

**Table 5:** Pharmacokinetic Parameters and Drug-likeness Based on Lipinski Parameters of the Synthesized Compounds

	physicochemical parameters						pharmacokinetic properties				
compounds P.K.	#stars	*M*_w_^a^	HBA	HBD	rotatable bond	PHOA	QPlog *P*o/w	*Ql*og *S*	QPPCaco	CNS^h^	violation of RO5
**5a**	1	448.30	4.75	1	4	78.64	5.505	–7.142	90.539	–1	0
**5b**	1	434.27	4.75	2	4	87.55	4.882	–7.148	43.471	–2	0
**5d**	1	452.72	4	1	3	93.35	5.834	–7.923	43.72	–2	0
**5c**	2	418.27	4	1	3	82.98	5.531	–7.28	89.126	–1	1
**5g**	1	428.82	5	1	4	66.58	4.199	–6.713	110.15	–1	1
**5e**	1	410.38	5.75	2	5	62.64	3.273	–5.993	10.63	–2	0
**5f**	0	424.40	5.75	1	5	66.41	3.925	–6.128	5.198	–2	0
**4o**	0	301.34	4	1	4	92.73	3.719	–4.935	7.342	–2	0
**4g**	0	347.36	5.5	1	6	91.71	3.895	–5.099	14.559	–2	0
**4k**	0	305.30	4	1	4	91.13	3.726	–4.896	16.218	–2	0
**4d**	0	321.76	4	1	4	89.70	3.977	–5.27	115.592	–2	0
**4e**	0	312.32	5.5	1	5	70.57	2.66	–5.316	157.905	–2	0
**4b**	0	317.34	4.75	1	5	87.76	3.587	–4.768	132.788	–1	0
**4p**	0	332.31	5	1	5	65.72	2.784	–4.69	130.961	–1	0
**4a**	0	287.31	4	1	4	84.61	3.256	–4.673	24.305	–2	0
**4l**	0	321.76	4	1	4	87.103	3.999	–5.264	15.591	–2	0
**4c**	0	305.30	4	1	4	87.37	3.765	–4.955	151.381	–1	0
**4h**	0	363.41	4	1	5	86.60	4.585	–5.91	68.146	–2	0
**4m**	0	293.33	4	1	4	85.62	3.323	–4.193	155.256	–1	0
**4i**	0	337.37	4	1	4	93.04	4.406	–5.544	130.692	–1	0
**4n**	1	390.65	4	1	4	96.06	4.932	–6.408	177.173	–1	0
**4f**	1	330.29	5	1	6	67.27	2.695	–4.522	131.241	–2	0
indomethacin	0	357.79	5.75	1	4	91.87	4.267	–5.122	162.164	–1	0

(HBA: Hydrogen-bond acceptors, HBD: Hydrogen-bond donors, *M*_w_: Molecular weight g/mol (ideally ≤500), PHOA: Percent Human Oral Absorption, QPlog *P*o/w: apparent permeability of compound between octanol/water, CNS^h^ activity in the central nervous system, QPPCaco: permeability of the differentiated cells of intestinal epithelium Caco-2, Qlog *S*: aqueous solubility (highly soluble > 0 > very soluble > – 2 > soluble > – 4 > moderately soluble > – 6 > poorly soluble > – 10 > insoluble), RO5: rule of five).

## 3 Evaluation of Computational Studies

### 3.1 Validation of Docking Protocol

Earlier, the lack of human COX-1 structure in the Protein Data Bank required homology modeling.^36^ However, a recent breakthrough (PDB ID: 6-Y3C) provided the crystal structure of homosapien COX-1.^37^ For COX-2 selectivity studies, the crystal structure of COX-2 complexed with salicylic acid (PDB entry: 5-F1a, resolution 2.38 Å) was used as the receptor. The docking process was validated by repositioning the ligand in COX-2 (5-F1a) and comparing it with the original complex, resulting in a root-mean-square deviation (RMSD) of 0.1857 Å. The overlap between the ligands in both structures confirms the reliability of the docking predictions. The redocking process was further validated by comparing calculated (Δ*G* = −7.467 kcal/mol) and experimental (Δ*G* = −7.58 kcal/mol) affinity values. The excellent agreement between these values indicates an accurate prediction of the interaction. Additionally, the RMSD value (less than 2 Å) reflects a high similarity between predicted and experimental binding modes of the 5-F1a proteins.^38^ These findings affirm the reliability of the redocking protocol for binding site prediction and support the biological significance of the 5-F1a proteins (Figures S89–S90)

### 3.2 Evaluation of Induced-Fit Docking Protocols

**Figure 6:**
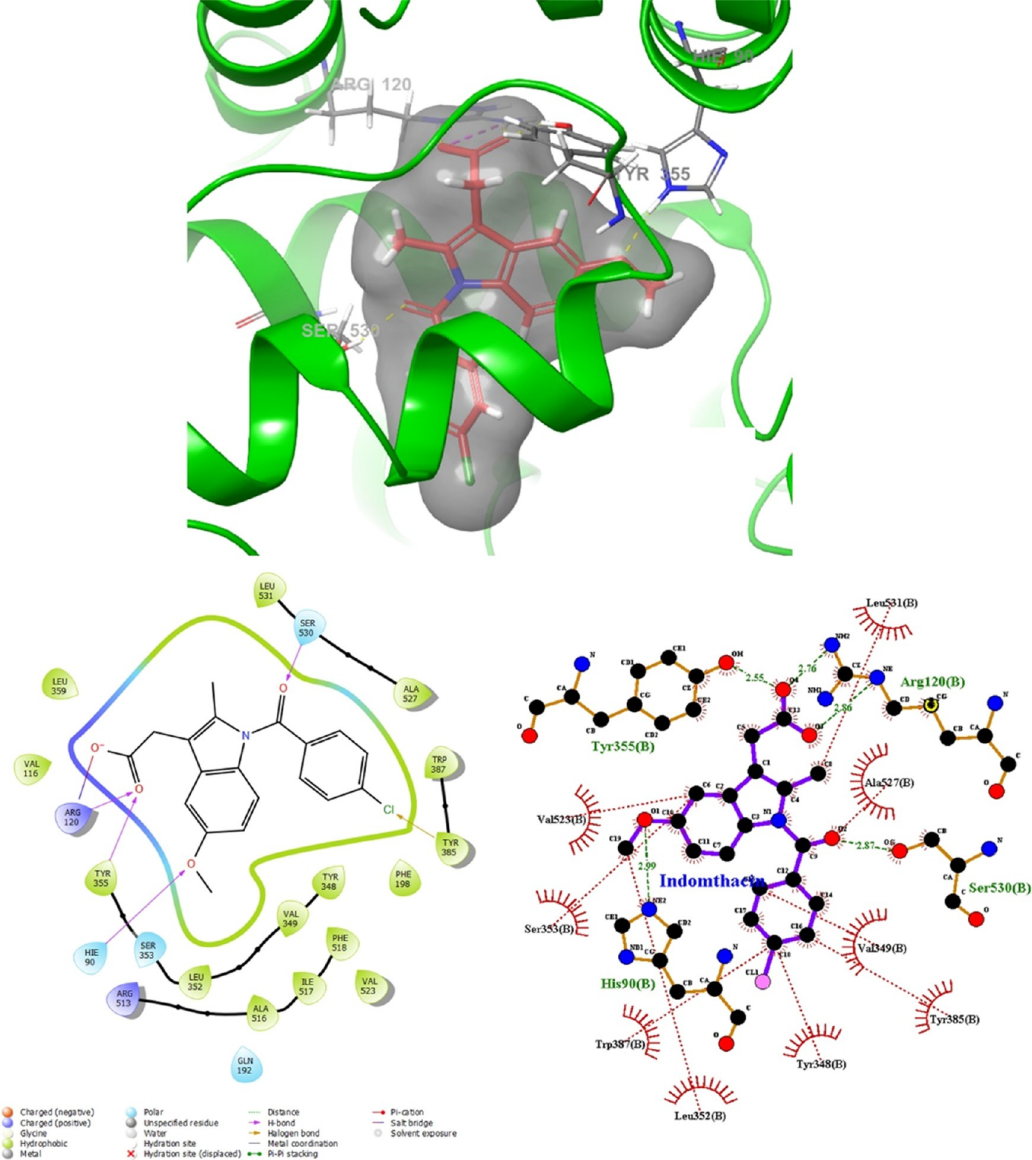
Docked pose of indomethacin in the binding site on the stem region (PDB ID: 5F1a) induced-fit docking (docking score:-11.380 kcal/mol).

We validated the induced-fit docking (IFD) protocol using indomethacin as the reference ligand for our synthesized compound. In the IFD simulation, indomethacin exhibited additional interactions, such as a hydrogen bond with His90 and distribution of the acyl aryl group throughout the binding site (Figure 6). These results highlight the superiority of the IFD protocol in capturing enhanced molecular interactions and conformational changes.

**Figure 7:**
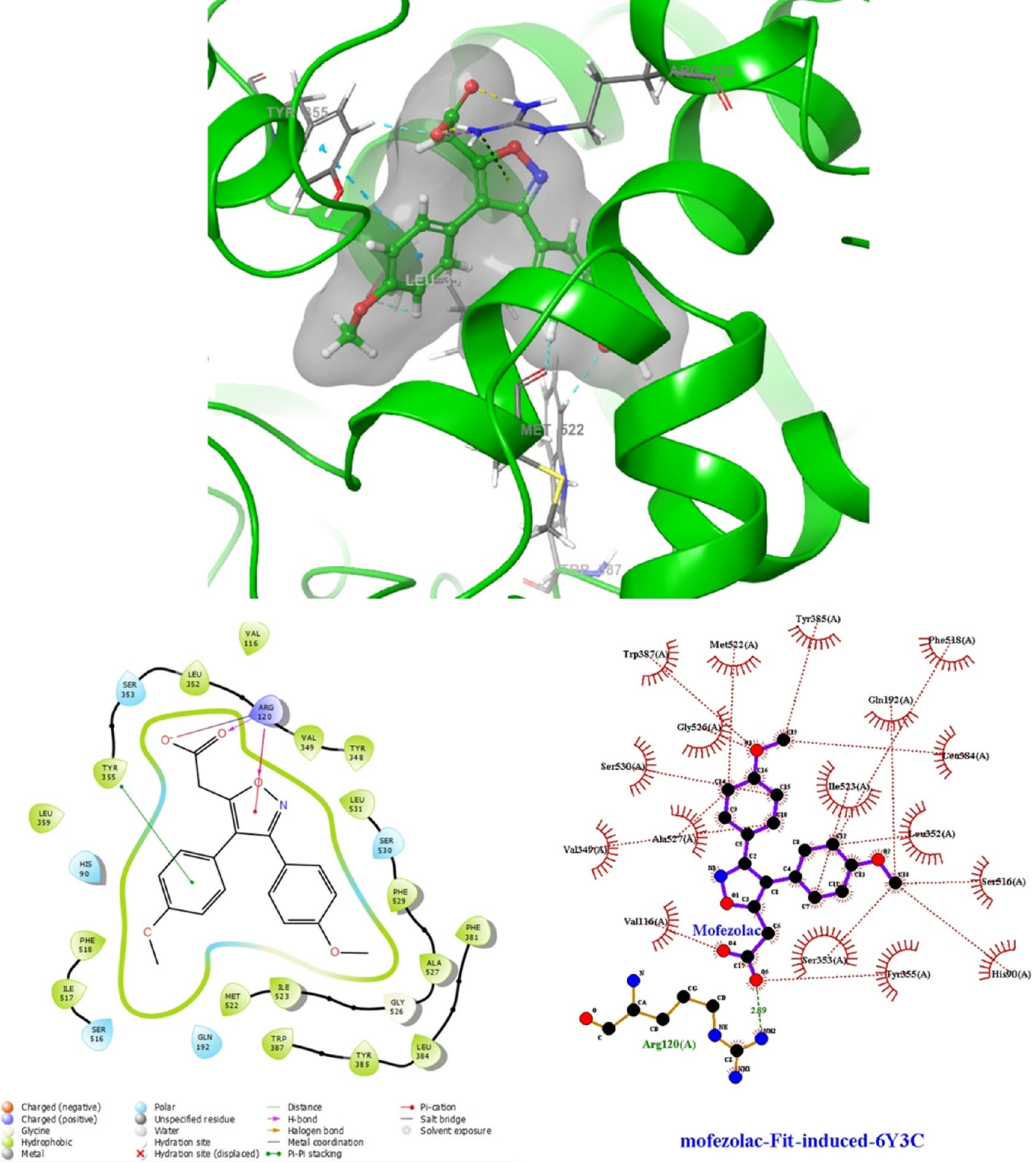
Docked pose of Mofezolac in the binding site on the stem region (PDB ID: 6-Y3C) induced-fit docking (docking score: -9.324 kcal/mol).

For the 6-Y3C PDB protein, where no crystal structure with a bound ligand was available, we employed IFD to predict Mofezolac’s binding mode (Figure 7). IFD revealed significant conformational changes in the protein upon ligand binding, establishing new interactions with Mofezolac. The interactions involved key residues such as Arg120 and Tyr355, indicating Mofezolac’s higher affinity for COX-1 over COX-2. However, it lacked interaction with Arg513, indicating its lack of selectivity toward COX-2 and preference for COX-1 inhibition.

### 3.3 Exploring In Silico Studies of Highly Active Compounds

#### 3.3.1 Analysis of Molecular Docking

**Figure 8:**
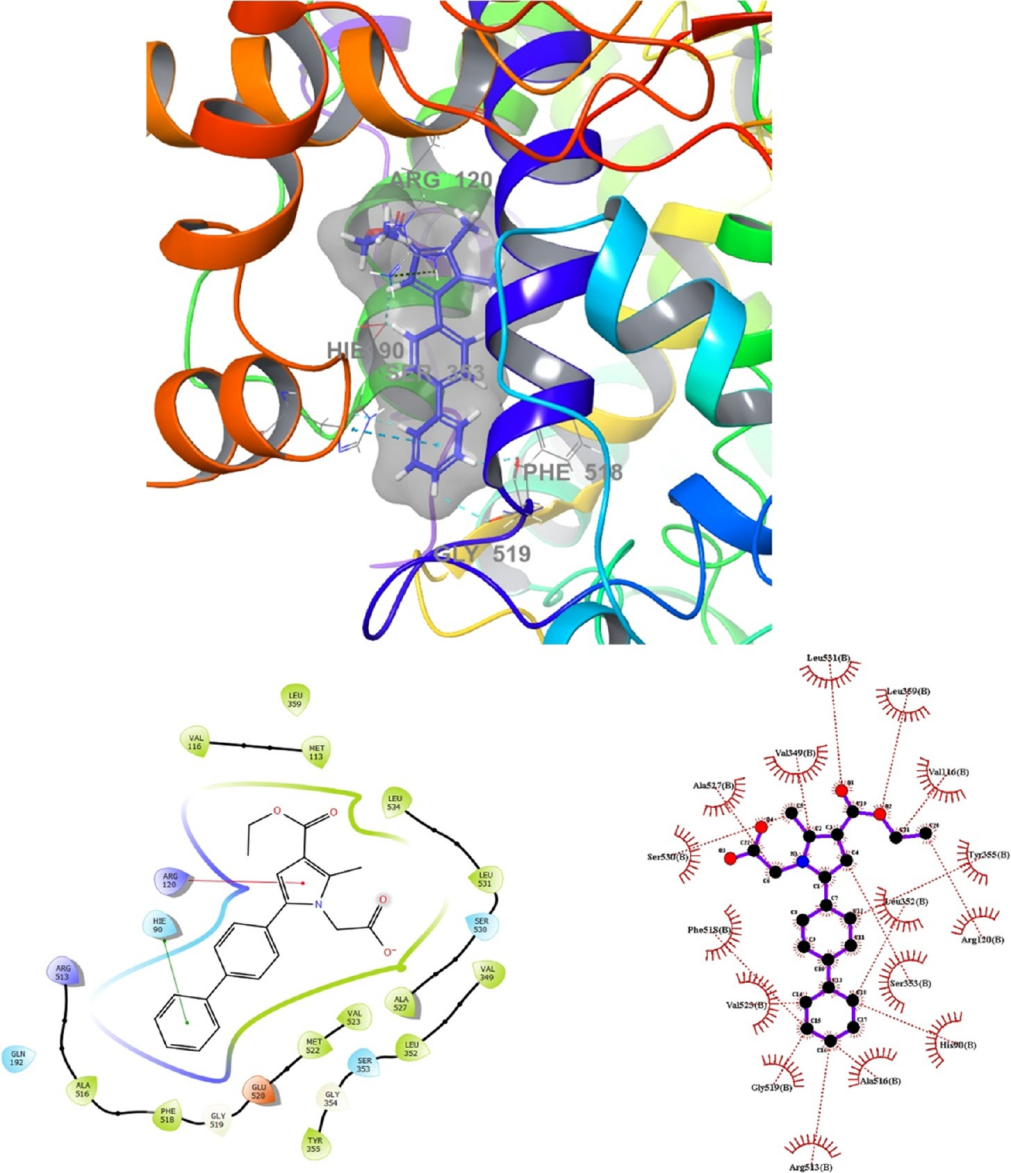
Superimposition pose and 2D interacting mode of **4h** in the active region of COX-2 (5-F1a).

**Figure 9:**
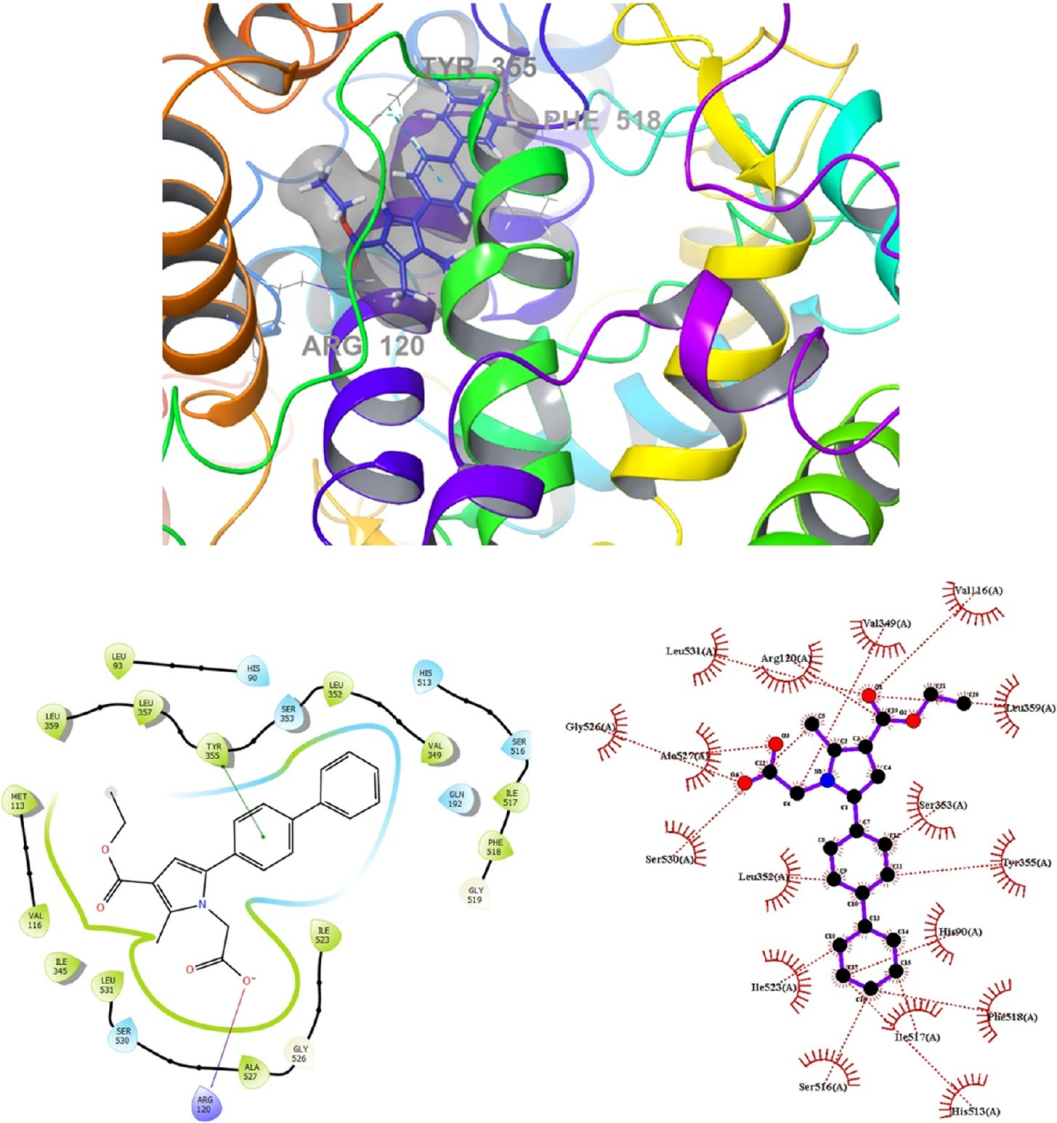
Superimposition pose and 2D interacting mode of **4h** in the active region of COX-1 (6-Y3C).

**Figure 10:**
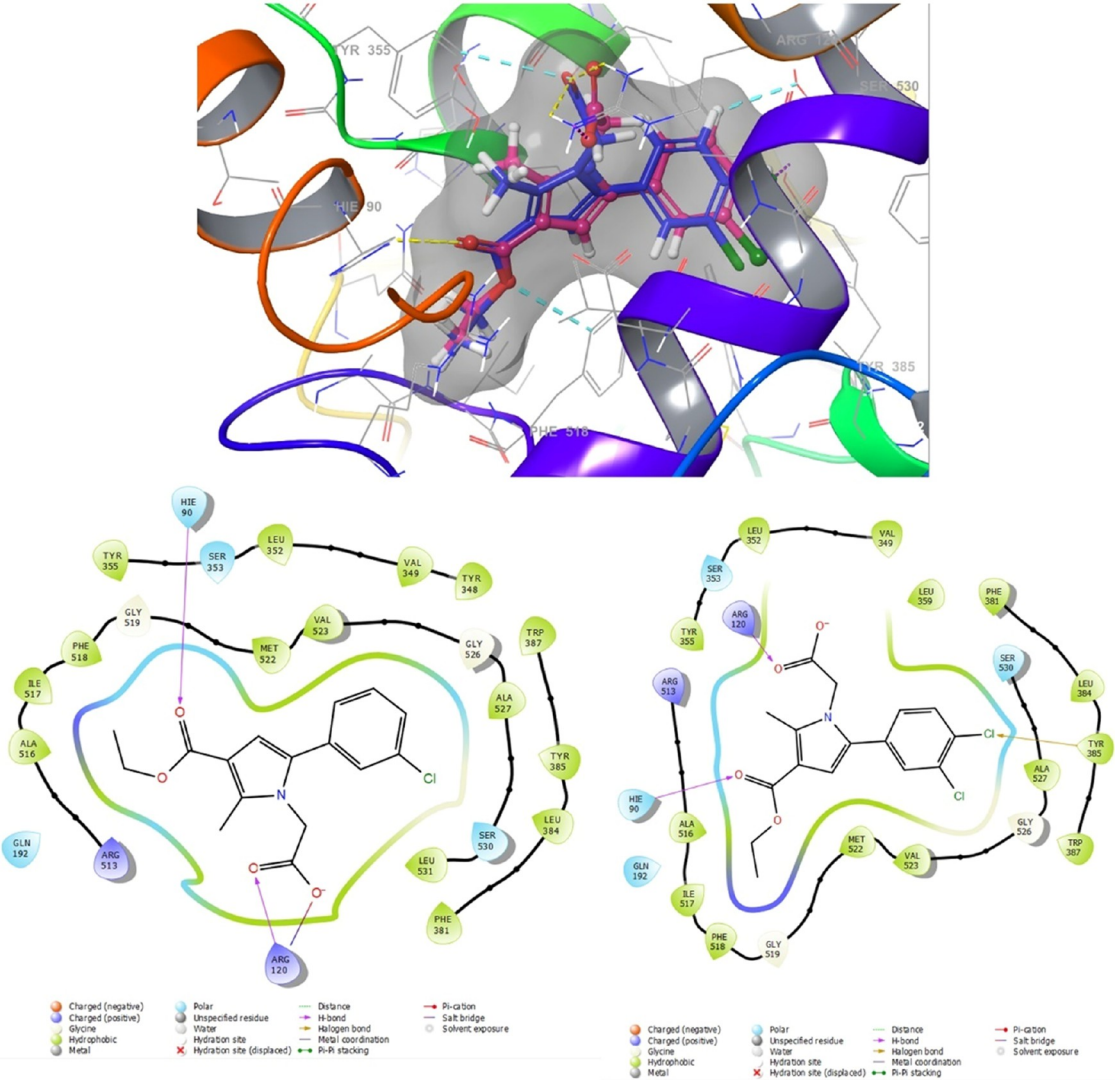
Superimposition pose and 2D interacting mode of **4k** and **4l** in the active region of COX-2 (5-F1a).

**Figure 11:**
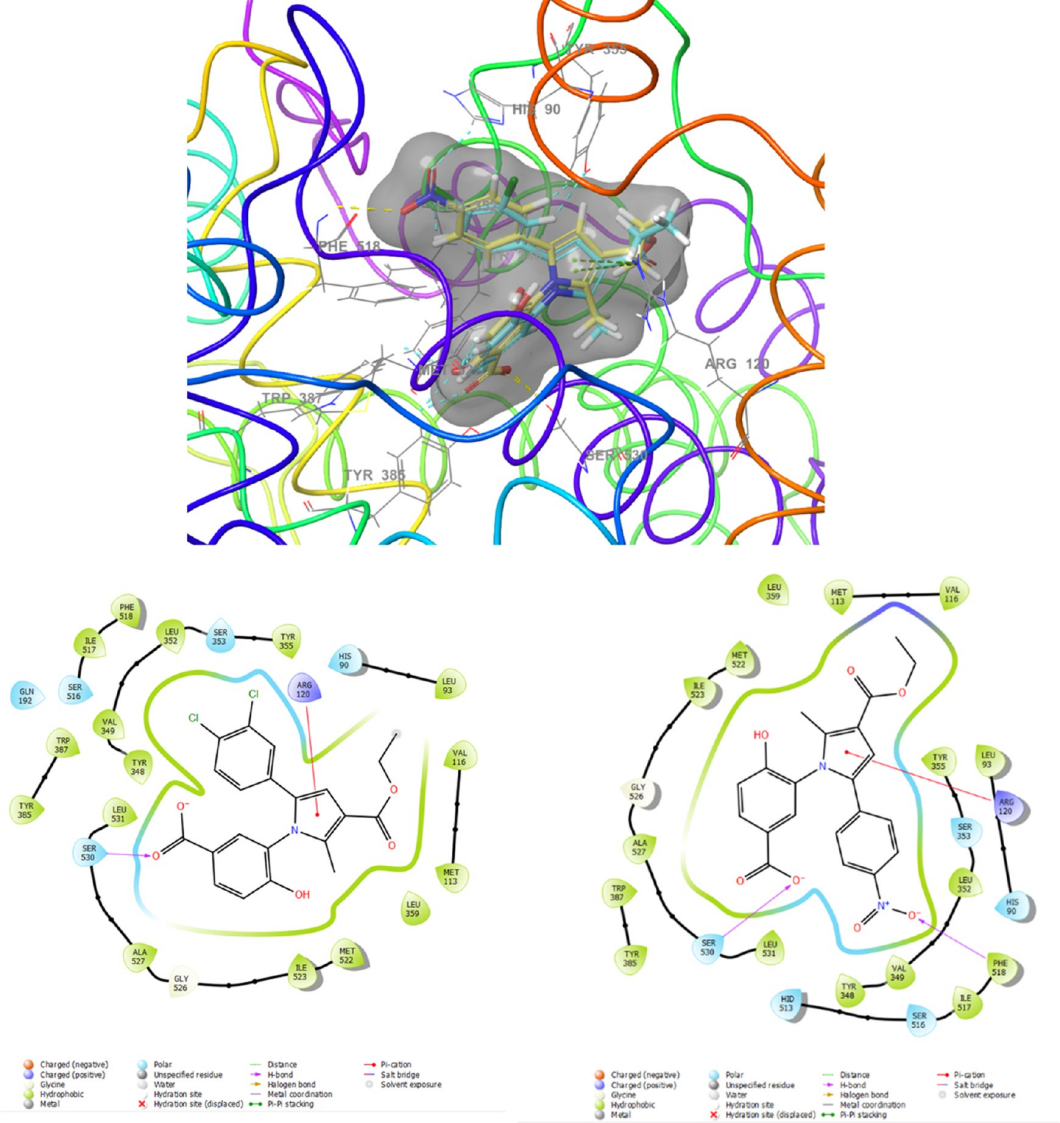
Superimposition pose and 2D interacting mode of **5e** and **5b** in the active region of COX-1 (6-Y3C).

Molecular docking simulations were conducted to predict the modes of binding of the synthesized compounds to the primary binding sites of both enzymes. The results are depicted in Figures 8, 9, 10, and 11. Compounds **4g** and **4h**, which displayed significant dual COX-1/COX-2 inhibitory activity in the biological assay, were chosen for docking studies. Using GLIDE software, these compounds were docked into the active sites of COX-1 and COX-2. While these compounds were categorized as “COX-2-favoring inhibitors” similar to meloxicam, etodolac, and nimesulide, their inhibitory activities and binding energy toward COX were relatively lower compared to Celecoxib. Notably, compound **4h** exhibited superior biological activity compared to Celecoxib (Table 3). This increased activity can be attributed to its unique ability to simultaneously interact with Arg120 and His90 while effectively occupying the lipophilic pocket with its biphenyl group, resulting in an enhanced binding affinity (refer to Figure 8). Furthermore, docking studies of **4h** in the COX-1 (6-Y3C) protein revealed interactions with Arg120 and Tyr355 (as shown in Figure 9). The presence of the biphenyl group significantly contributed to enhancing the activity of both COX-1 and COX-2. Additionally, the inclusion of small and electrostatic groups at positions 1 and 3 facilitated the occupation of the biphenyl moiety within the active site, enabling interactions with crucial amino acids essential for COX-1 and COX-2 activity. Figure 10 presents the docking results, indicating that the benzene ring and ethoxycarbonyl group of compounds **4k** and **4l** adopted a similar orientation and were situated within two of the three key binding pockets of the COX-2 binding site, suggesting their potential as COX-2 inhibitors. The acetic acid pyrrole derivatives, **4k** and **4l**, containing 3,4-dichlorophenyl and 3-chlorophenyl moieties at position 5, respectively, exhibited orientation similarities to the cocrystallized inhibitor, indomethacin. However, the carbonyl on the ethoxycarbonyl group of **4k** and **4l** formed hydrogen-bonding interactions with His90, whereas the methoxy group in indomethacin established the same interaction with this residue. Additionally, the carboxyl group at position 1 of **4k** and **4l** and position 3 of indomethacin formed a salt bridge and hydrogen bond interactions with Arg120. The methyl substituent at position 2 of the pyrrole ring occupied a polar pocket between the side chains of Arg120 and His90. The corresponding 3-hydroxybenzoic acid derivatives, compounds **5e** and **5b**, exhibited a shift in activity toward COX-1 due to different interactions formed by the carboxyl group on the benzoic acid and the substituted phenyl group at position 5. The pyrrole ring interacted through a Pi-cation interaction with Arg120, while the substituted phenyl group occupied the hydrophobic pocket similar to COX-2 inhibitors. Compound **5e**, containing a nitro group, formed a hydrogen-bonding interaction with Phe518, while both phenyl groups in **5e** and **5b** established asymmetric hydrogen bonds with Tyr355, His90, and Leu352 (Figure 11).

Overall, compounds **4h**, **4k**, and **4l** exhibited significant COX-1/COX-2 inhibitory activity and demonstrated a high potential for effective binding with the COX-2 enzyme, making them potential candidates for the development of new selective COX-2 inhibitors. Although they were considered “preferential COX-2 inhibitors”, their inhibitory activities and binding energy were noteworthy compared to highly selective COX-2 inhibitors like Celecoxib. These findings provide insights into the molecular mechanisms underlying COX-1/COX-2 inhibition and suggest promising directions for the development of more effective anti-inflammatory drugs.

#### 3.3.2 In-Depth FB-QSAR Analysis

To identify the most active compounds, we conducted in vivo experiments and synthesized several compounds with diverse chemical structures. Subsequently, we utilized the FB-QSAR model to predict the activity of these compounds. Interestingly, the model accurately predicted the activity of the most potent compound identified in the in vivo studies, as mentioned in Section 2.3, demonstrating its reliability and usefulness. Our plans involve employing the FB-QSAR model to predict the activity of additional compounds with modified chemical structures that have been synthesized. The insights obtained from this model will deepen our understanding of the factors influencing the activities of COX-1 and COX-2 inhibitors and facilitate the rational design of novel and more potent inhibitors with improved selectivity and therapeutic effectiveness.

**Figure 12:**
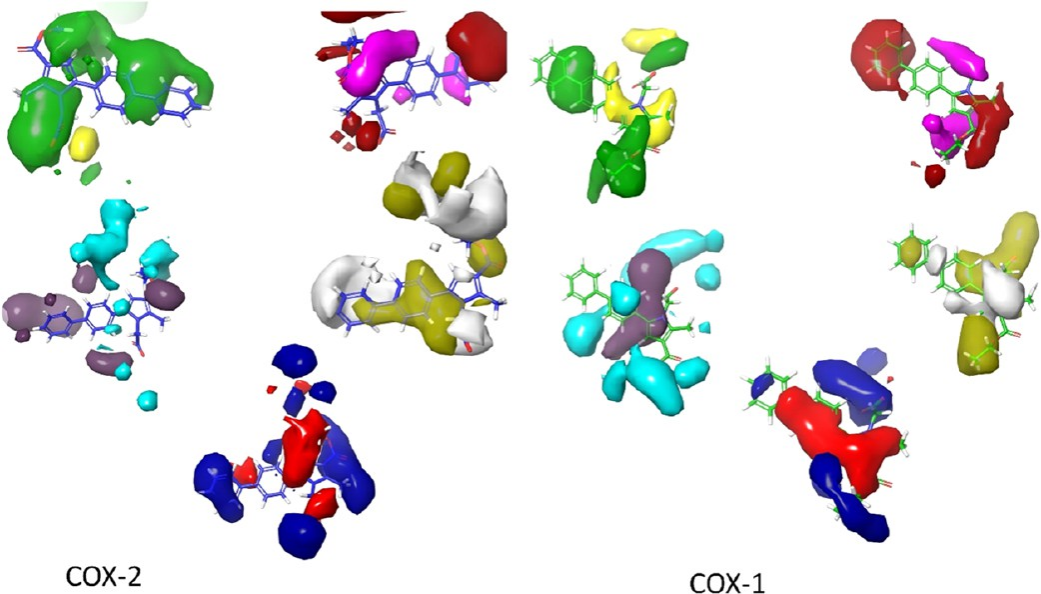
3D visualizations of **4h** contour maps COX-2 & COX-1: steric (positive effect (+): green, negative effect (_): yellow), electrostatic (+: blue, -: red), hydrophobic (+: yellow, -: white), HBA (+:red, -:magenta), and HBD (+:blue-violet, -: cyan), respectively.

The findings revealed that positioning a biphenyl ring at position 4 with small lipophilic groups at position 1, instead of potential hydrogen-bond acceptor (HBA) or hydrogen-bond donor (HBD) groups, resulted in increased activity. Furthermore, the replacement of the terminal phenyl group with a bioisosteric substitute such as pyridine resulted in improved electrostatic interactions in that specific area, as evidenced by the positive electrostatic and HBA contour maps, which contributed to the enhanced activity toward COX-2 and COX-1, respectively (see Figure 12). The contour map of the acetic acid group indicated the presence of a small lipophilic group, aligning with the electrostatic interaction observed in the FB-QSAR-generated contour map. The small methyl group in the acetic acid should not possess any HBA or HBD groups either.

Although the biphenyl substitution may act as a steric hindrance group, potentially impeding the compound from entering the smaller COX-1 pocket compared with COX-2, the biological activity demonstrated that compound **4h** exhibited good activity in COX-1. This suggests that the presence of the biphenyl group facilitates the compound’s flat or planar shape, allowing easy access to the COX-1 active site. Consequently, this QSAR model can be utilized to identify new and existing pyrrole carboxylic acid derivatives with COX-2 inhibition activity. Moreover, it can serve as a valuable intermediary model for developing new QSAR hypotheses for dual inhibitors targeting both COX-1 and COX-2 enzyme isomers.

#### 3.3.3 Molecular Dynamics Simulation

**Figure 13:**
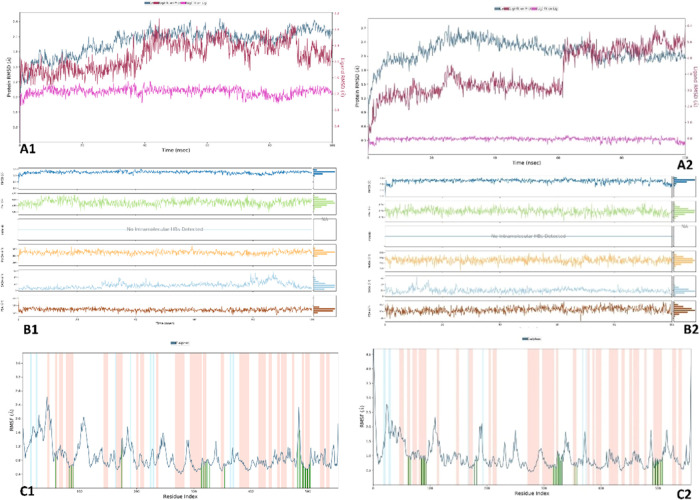
Stability diagrams and ligand properties (RMSD, *R*_g_, MolSA, SASA, PSA) and RMSF analysis of complexes **4h**, 6-Y3C-complex (**A2, B2, C2**) and 5-F1a-complex (**A1, B1, C1**).

**Figure 14:**
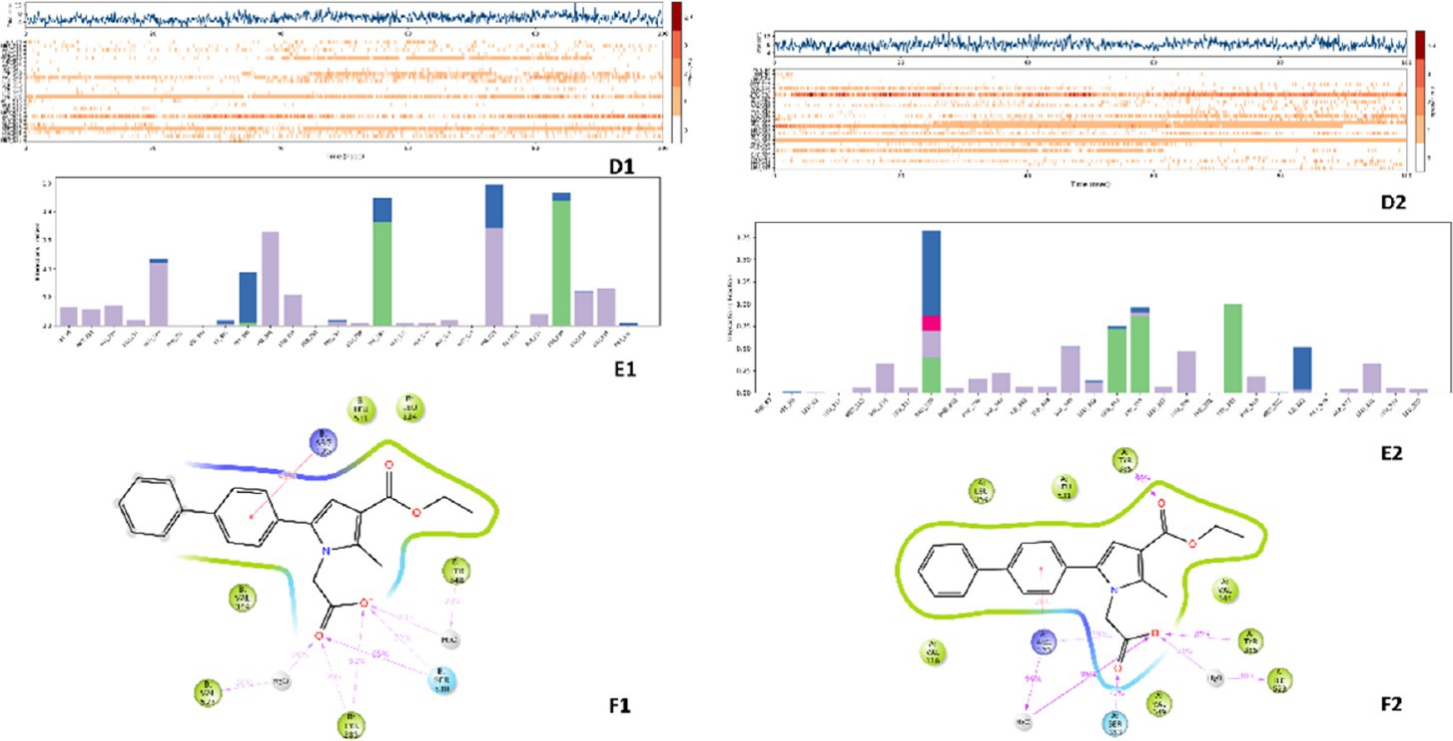
Interaction diagrams of the complexes of 5-F1a (left) and 6-Y3C (right). **D1, D2**: The plot of total bond number-amino acid fraction during the simulation time. **E1, E2**: Types of interactions with the amino acids and their fraction graphic. **F1, F2**: The bond strength (cutoff = 20%), respectively.

The aim of this analysis was to evaluate the stability and structure–activity relationship (SAR) of the **4h** ligand within the 6-Y3C and 5-F1a protein complexes. The stability of the complexes was assessed using molecular dynamics simulation (MDS), and the results, as depicted in Figure 13, indicated that both complexes exhibited values within an acceptable range. The simulations also revealed that the *R*_g_ plots showed no significant changes, suggesting the overall stability of the complexes. The RMSD values for COX-1 and COX-2 remained below 3 Å, indicating minimal fluctuations in key structural elements such as the Å-helix (red areas) and Å-strand (blue areas). Overall, the complexes demonstrated a stabilizing effect on their structures. Further examination using RMSF plots highlighted the positive impact of interactions with loop amino acids (green line in the white area), resulting in a reduced intensity of fluctuations. This finding indicates that both complexes effectively maintained stable ligand–protein interactions throughout the simulation. After confirming complex stability, Figure 14 presents an evaluation of the types, continuity, and strength of interactions. In both protein complexes, hydrophobic interactions were observed between **4h** and specific amino acid residues, including His90, Met113, Val116, Leu117, Arg120, Ile345, Val349, Leu352, Tyr355, Leu359, Arg513, Arg518, Val523, Ala527, Leu531, and Leu534. Additionally, the 5-F1a protein formed hydrogen bonds (HBs) with Tyr348, Tyr385, and Ser530, while the 6-Y3C protein formed HBs with Arg120, Tyr355, Tyr385, and Ser353. Water-mediated hydrogen bonds were detected in the 5-F1a complex with Arg120, Ile345, Tyr348, Tyr355, Tyr385, Val523, Ser530, and Met535, while in the 6-Y3C complex, they were observed with His90, Arg120, Leu352, Ser353, and Ile523. Furthermore, ionic interactions were noted with Arg120 in the 6-Y3C complex. Notably, the ligand exhibited strong interactions with specific residues, including Arg120, Tyr248, Val349, Tyr385, Val523, and Ser530 in the 5-F1a complex and Arg120, Ser353, Tyr355, and Tyr385 in the 6-Y3C complex. Videos S1 and S2 focus on highlighting aromatic hydrogen bonds (AHBs) and hydrophobic interactions involving the 5-F1a protein complex. These interactions were visually represented by faded teal and blue dashes, respectively, while other types of interactions were hidden to enhance clarity. Notably, AHBs, hydrogen bonds (HBs), ionic interactions, and water-mediated HBs were observed between the **4h** ligand and specific amino acids, such as Ty355 and Arg120. These interactions indicate the stability of the bonds within the active site without any breakage or loss. The analysis of interaction continuity revealed that in the 5-F1a complex, the interaction between **4h** and Arg120 exhibited an H-bond strength of 43%, while in the 6-Y3C complex, the corresponding interaction with Arg120 displayed a higher H-bond strength of 96%. Additionally, in the 6-Y3C complex, the interactions between **4h** and Tyr385, Tyr355, and Ser352 showed interaction strengths of 99, 85, and 71%, respectively. These findings provide insights into the persistence and strength of the interactions between **4h** and specific residues within the complexes.

### 3.4 Evaluation of the Structure–Activity Relationship

**Figure 15:**
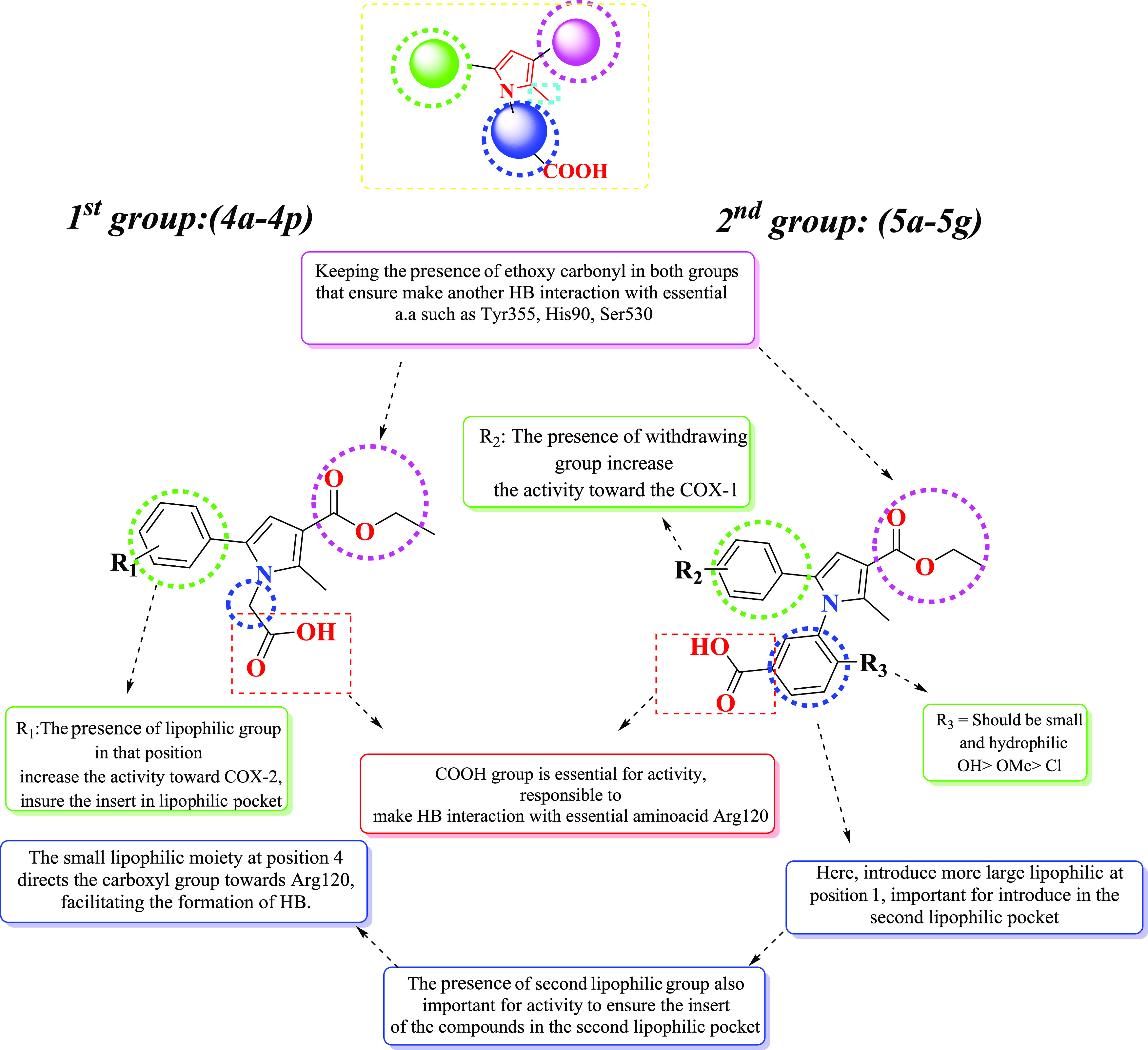
Summary of the structure–activity relationship of synthesized compounds.

To investigate the structure–activity relationship of our synthesized compounds, we conducted in vivo assays, utilized FB-QSAR, and performed molecular docking studies. Our findings underscore the crucial role of carboxylic acid in the activities of the compounds. This is attributed to its ability to form hydrogen bonds and salt bridges with essential amino acids, including Arg120, Arg513, Tyr385, His90, Ser353, and Ser530, within the active sites of the COX-1 and COX-2 enzymes. Specifically, the acetic acid group at position 1 forms a hydrogen bond with Arg120, while the benzoic acid group at the same position forms a hydrogen bond with Ser530. Consequently, we observed that compounds with benzoic acid groups exhibited higher efficacy in COX-1, whereas those with acetic acid groups demonstrated greater efficacy in COX-2. Additionally, introducing a withdrawing group on the phenyl ring at position 5 led to reduced activity against both COX-1 and COX-2, as evident in compounds **4f** and **4g**. Moreover, our findings revealed that incorporating a hydroxy group at position 4 of hydroxybenzoic acid significantly enhanced the potency of the compounds against COX-1, as observed in compounds **5b** and **5e**. This increased potency can be attributed to the additional opportunity for the formation of hydrogen-bond interactions within the COX-1 active site. Conversely, when larger and more lipophilic groups such as OMe and Cl were introduced, as seen in compounds **5a**, **5f**, **5d**, and **5g**, they engaged in hydrophobic interactions within the active site. Our investigation further revealed that the most active compounds featured large hydrophobic substitutions on the phenyl ring, such as phenyl substitution at position 4 in compound **4h**, OMe substitution at positions 2 and 6 in compound **4g**, Cl substitution at positions 3 and 4 in compound **4k**, and the naphthyl group in compound **4l**. These compounds exhibited greater effectiveness in COX-2 compared to COX-1. In contrast, withdrawing groups at position 4, such as NO_2_ in compound **5e** and Cl group in compound **5b**, rendered the compounds more effective in COX-1, likely due to their retention of hydrophobic characteristics in the presence of benzoic acid substitution at position 1. Our study provides valuable insights into the factors influencing the COX-2 inhibitor activity, facilitating the rational design of more potent inhibitors with improved selectivity and therapeutic efficacy. The summarized structure–activity relationship (SAR) of the synthetic compounds is presented in Figure 15.

## 4 Conclusions

The present study involves the development of novel compounds based on pyrrole nucleus. This study introduces novel pyrrole-based compounds targeting inflammation by inhibiting the enzyme cyclooxygenase (COX) responsible for prostaglandin production. Through FB-QSAR, diverse alkyl and aryl carboxylic acid derivatives were synthesized and analyzed using HRMS, ^1^H NMR, and ^13^C NMR. Compound screening based on QSAR parameters identified active molecules, including nonselective inhibitors (**4g, 4h**), selective COX-1 inhibitors (**5e, 5b**), and selective COX-2 inhibitors (**4k, 4l**). Structural modifications influenced the selectivity of the COX subtype selectivity. Molecular docking and dynamic simulations provided insights into binding modes and structure–activity relationships. This research holds promise for novel anti-inflammatory agents and offers valuable methodologies for the design of bioactive compound design.

## 5 Materials and Methods

### 5.1 Preparation of Database Library and Development of FB-QSAR Model

To assess the effectiveness of our filters and understand their impact on the chemical landscape, a rigorous evaluation was necessary. We utilized the ChEMBL database (https://www.ebi.ac.uk/chembl/; accessed date: July 10, 2022) as our primary resource. From this database, we obtained identifiers 4275 and 9521 for COX-1 and COX-2, respectively (CHEMBL code: CHEMBL 221, and CHEMBL 230).

We focused on compounds with IC_50_ values (log IC_50_ ≥ 6.00) and applied a ligand filter in Maestro to narrow the selection. This filter specifically identified compounds containing tetra-substituted pyrrole motifs, resulting in the identification of 477 and 290 compounds for COX-2 and COX-1, respectively. To further analyze these compounds, we performed docking and minimization using the Glide plugin in Maestro. Subsequently, the compounds were imported into the three-dimensional (3D) field-based QSAR interface for in-depth investigation. The training set was set to 75%, and the selection was made randomly. The FB-QSAR model was developed using default settings, employing a Partial Least Squares (PLS) factor of 5. To evaluate the reliability and validity of the FB-QSAR model, various parameters, including *R*^2^, *Q*^2^, Pearson correlation coefficients (Pearson^–r^), stability, RMSE, *F*, and *P* values, were examined.^15^ These parameter thresholds were validated based on specific criteria, such as maintaining a dissimilarity value of less than 0.1 between *R* and *Q*, and requiring Pearson correlation coefficients (|*r*_c_|) to have an absolute value of 0.3 or higher.^16^ The results obtained from the FB-QSAR model were then utilized to guide the design and synthesis of our targeted compounds. By leveraging the insights gained from the model, we aimed to develop novel compounds with enhanced properties and improved efficacy.

### 5.2 Chemistry

All chemicals used in the syntheses were purchased from either Merck Chemicals (Merck KGaA, Darmstadt, Germany) or Sigma-Aldrich Chemicals (Sigma-Aldrich Corp., St. Louis, MO). The reactions and purities of the compounds were observed by thin-layer chromatography (TLC) on silica gel 60 F254 aluminum sheets obtained from Merck (Darmstadt, Germany). Melting points of the synthesized compounds were recorded by an MP90 digital melting point apparatus (Mettler Toledo, Ohio) and were presented as uncorrected. ^1^H NMR and ^13^C NMR spectra were recorded by a Bruker 300 and 75 MHz digital FT-NMR spectrometer (Bruker Bioscience, Billerica, MA, USA) in DMSO-*d*_*6*_, respectively. In the NMR spectra, splitting patterns were designated as follows: s: singlet; d: doublet; t: triplet; m: multiplet. Coupling constants (*J*) were reported as hertz. High-resolution mass spectrometric (HRMS) studies were performed by using an LC/MS-IT-TOF system (Shimadzu, Kyoto, Japan). Elemental analyses were performed on a Leco 932 CHNS analyzer (Leco, Michigan).

#### 5.2.1 Method A: Synthesis of Ethyl Acetoacetate Sodium Salt (**1**)

The ethyl 3-oxobutanoate sodium salt **(1)** was made by reacting an equal amount of acetoacetate ethyl ester (2.8 mL, 21.90 mmol) with metallic sodium (0.5 g, 21.7 mmol) at 0 °C for 5 days in the presence of anhydrous toluene.

#### 5.2.2 Method B (Bromination): Synthesis of Substituted Bromo-Acetophenones (**2**)

For the bromination step, a solution of bromine (1.2 equiv) in glacial acetic acid was added drop by drop to a stirred solution of substituted acetophenones in glacial acetic acid with just two drops of hydrobromic acid for 6–8 h at 0 °C.

#### 5.2.3 Method C: Synthesis of Ethyl 2-acetyl-4-oxo-4-(substituted phenyl) Butanoate (**3**)

Using substituted bromoacetophenones **(2)** as the nucleophilic reagent, the salt ethyl acetoacetate sodium salt **(1)** is transformed into compound **(3)**. The toluene used as a solvent was evaporated after the reaction was finished. The residue was washed with water and filtered to create the desired output.

#### 5.2.4 Method D: Synthesis of 2-[3-(Ethoxycarbonyl)-2-methyl-5-(substituted phenyl)-1*H*-pyrrol-1-yl]substituted Carboxylic Acid (**4a**–**4p** and **5a**–**5g**)

The pyrrole ring was closed under Paal–Knorr conditions by refluxing equimolar amounts of amino derivatives (2 equiv) and ethyl 2-acetyl-4-oxo-4-(substituted phenyl) butanoate (**3**) in glacial acetic acid for 12–24 h. The reaction was monitored using TLC, the reaction was worked up using iced water, and the precipitated product was extracted using ethyl acetate (Supporting Data)

### 5.3 Determination of COX-1 and COX-2 Inhibitors Activity

To conduct the inhibition assay, the kit components were prepared as follows: The COX-1/COX-2 enzyme solutions were created by reconstituting the lyophilized powder with 110 μL of ddH_2_O. A diluted COX cofactor was formed by combining a COX assay buffer (398 μL) and a COX cofactor (2 μL). The arachidonic acid/NaOH solution was diluted with 5 μL of arachidonic acid, 5 μL of NaOH, and 90 μL of ddH_2_O. These solutions were combined to generate the reaction mixture (80 μL) for each well, consisting of the COX assay buffer (76 μL), the COX probe (1 μL), the diluted COX cofactor (2 μL), and the COX-1/COX-2 enzyme solution (1 μL). Test compounds (10 μL) were added, followed by incubation for 5–10 min at 25 °C. To stop the reaction, 10 μL of diluted arachidonic acid/NaOH solution was added. The fluorescence of the samples was measured kinetically at 5 min intervals using a BioTek-Synergy H1 multimode microplate reader (BioTek Instruments, Inc., Winooski, VT) at an excitation/emission wavelength of 535/587 nm. The assay was performed in quadruplicate with a blank and control, testing all inhibitor concentrations. The mean ± standard deviation (SD) of the percentage inhibition results were calculated, and IC_50_ values were determined using GraphPad PRISM software (version 5.0) by plotting the percentage inhibition against the log concentration. Additionally, the selectivity index (SI) was calculated by dividing IC_50(COX-1)_ by IC_50(COX-2)._

### 5.4 Computational Studies

#### 5.4.1 Molecular Docking

In our computational studies, we aimed to understand interactions between inhibitors and COX-1 and COX-2 proteins, seeking novel COX-1 and COX-2 inhibitors (6-Y3C and 5F1a, respectively). We chose homosapien proteins,^17^ and validated crystal structures from the PDB (www.pdb.org). COX-2 has a larger binding cavity than COX-1, and its unique side pocket enables selective targeting.^18^ Discoveries regarding the Arg513 and Leu384 positions offer valuable insights for designing novel COX-2 inhibitors.^19^ Understanding these features aids in designing specific COX-1 and COX-2 inhibitors with potential therapeutic advantages.^19^

In our previous studies, we refined the structures of COX-1 and COX-2 proteins using Maestro’s Protein Preparation Wizard.^20,21^ Ligprep was then used to prepare the compounds at a physiological pH with an OPLAS4 force field. Minimization with the PRCG algorithm and a conformational search ensured dependable and precise docking results.^22^

We used induced-fit molecular docking (IFD) in this study to accurately predict how the ligand would bind to the COX-1 and COX-2 proteins. IFD considers multiple factors, ensuring reliable and precise docking results. It allows the ligand to dock into the binding site, guesses how the ligand will conform within the protein, and takes into account electrostatic, flexible, and van der Waals interactions.^23^ IFD also takes into account how the structure changes when a ligand binds, which gives a more accurate picture of the important interactions in the protein–ligand complex.^24^ The Glide module^25^ was established based on the induced protein conformation, taking into account shape and new ligand interactions. The grid box (radius 20) centered at *X* = 18.1, *Y* = 51.98, and *Z* = 17.42 focused on key amino acid residues THR94, Tyr355, Tyr385, Ser353, Tyr348, and Ser530 in the COX enzymes. All docking runs were optimized using standard precision (SP) docking mode.

#### 5.4.2 Molecular Dynamics Simulation (MDS)

In our previous studies, alongside molecular docking, we conducted molecular dynamics (MD) simulations to assess ligand–receptor complex stability.^26^ MD simulations were run for 100 ns using the Desmond application.^27^Complex optimization, neutralization, and dynamics conditions were applied by a System builder application. The condition was applied as follows: 310.55 K, 1.01325 bar pressure using NPT ensemble. Neutralization was done with a NaCl solution. Important parameters like *R*_g_, RMSF, and RMSD were calculated using Desmond’s equation to understand structural dynamics and stability, enhancing our understanding of binding interactions. Analyzing the MD results aids in characterizing ligand–receptor complexes, which is crucial for developing novel drugs with improved binding affinity and selectivity.

#### 5.4.3 Prediction of Physicochemical, Drug-likeness, Pharmacokinetic, and Toxicokinetic Properties

The process of clinical trials and regulatory approval is rigorous, resulting in only a small fraction of compounds with optimal pharmacokinetic and toxicokinetic properties reaching the market.^28^ Poor bioavailability, characterized by limited absorption, rapid elimination, and hepatic clearance, presents challenges in drug development.^29^ Therefore, it is crucial to consider a compound’s absorption, distribution, metabolism, excretion, and toxicity (ADMET) descriptors in early drug development to assess safety and potential efficacy. In this study, the ADMET profiles and drug likeliness of all compounds and standards were evaluated using Qikprop (Schrödinger, LLC, New York, NY). The compounds were assessed based on Lipinski’s Rule of Five, which defines criteria such as molecular weight (*M*_W_) ≤ 500 Da, ≤ 5 hydrogen-bond donors (HBD), ≤ 10 hydrogen-bond acceptors (HBA), and a log (octanol/water) partition coefficient (log *P*) < 5 to determine drug-likeness. Compounds violating more than one of Lipinski’s Rules of Five were excluded. Additionally, the number of allowable rotatable bonds was limited to between 0 and 15.^30^

## Supporting Information Available

Dynamics of the protein–ligand interactions (Video S1) (MP4)
Dynamics of the protein–ligand interactions (Video S2) (MP4)
Analytical data: analytical spectra including HRMS, ^1^H NMR, and ^13^C NMR, analytical results and InChI descriptions for the synthesized compounds, a comprehensive summary of the ^13^C NMR and ^1^H NMR chemical shift values for common scaffolds in targeted compounds; molecular docking and simulation data: detailed information on docking poses; molecular dynamics (MD) stimulation assembly details; a comparison between crystal and simulated poses of 6-Y3C and 5-F1a crystals, functional group-based quantitative structure–activity relationship (FB-QSAR) models; and molecular dynamics simulations (MDS): MDS of 6-Y3C and 5-F1a complex protein (ZIP)
